# Cardiac autoantibodies promote a fibrotic transcriptome and reduced ventricular recovery in human myocarditis

**DOI:** 10.3389/fimmu.2025.1500909

**Published:** 2025-03-20

**Authors:** Jennifer M. Myers, Clayton Sandel, Kathy Alvarez, Lori Garman, Graham Wiley, Courtney Montgomery, Patrick Gaffney, Stavros Stavrakis, DeLisa Fairweather, Katelyn A. Bruno, Yan Daniel Zhao, Leslie T. Cooper, Madeleine W. Cunningham

**Affiliations:** ^1^ Department of Microbiology and Immunology, University of Oklahoma Health Sciences Center, Oklahoma City, OK, United States; ^2^ Genes and Human Disease Research Program, Oklahoma Medical Research Foundation, Oklahoma City, OK, United States; ^3^ Clinical Genomics Core, Oklahoma Medical Research Foundation, Oklahoma City, OK, United States; ^4^ Department of Cardiology, University of Oklahoma Health Sciences Center, Oklahoma City, OK, United States; ^5^ Department of Cardiovascular Medicine, Mayo Clinic, Jacksonville, FL, United States; ^6^ Biostatistics and Epidemiology, University of Oklahoma Health Sciences Center, Oklahoma City, OK, United States

**Keywords:** myocarditis, transcriptomics (RNA sequencing), autoantibodies, cardiomyopathy, autoimmunity

## Abstract

Myocarditis leads to dilated cardiomyopathy (DCM) with one-third failing to recover normal ejection fraction (EF 50%). Our previous studies have supported a Th17 autoimmune pathogenesis where IL17A and IL-6 are elevated in myocarditis patients who do not recover normal EF. In the non-recovered group, autoantibody mechanisms of pathogenesis in myocardial injury and systolic dysfunction are not fully understood. Furthermore, in our myocarditis cohort, cardiac myosin (CM) autoantibodies (AAbs) were elevated and cross-reactive with the β−adrenergic receptor (βAR). Here we studied cross-reactive CM/βAR serum AAbs and human myocarditis-derived monoclonal antibodies (mAbs) to define their potential pathogenic mechanisms and to identify unique human CM epitopes associated with non-recovery in a longitudinal (n=41) cohort. Elevated CM IgG AAbs in the non-recovered phenotype correlated with reduced EF and poor outcomes. Human CM epitopes unique to the non-recovered phenotype shared strong amino acid sequence homology with extracellular loops of βARs and supported molecular mimicry and cross-reactivity between CM and βAR. Myocarditis-derived IgG and human mAb 2C.4 activated protein kinase A (PKA) in an IgG, CM, and βAR-dependent manner in H9c2 heart myoblast cell line, and transcriptomic analysis revealed mAb 2C.4 induced fibrosis pathways which were highly similar pathways seen with isoproterenol, a beta receptor agonist. Our data translate into new mechanistic insights from our small longitudinal group of myocarditis/DCM patients and into potential therapeutic targets and biomarkers for future studies.

## Introduction

Autoimmunity leading to heart disease is not well understood due to the heterogeneity of immune mediated heart diseases as well as the lack of knowledge about specific immune mechanisms in the heart. Myocarditis is a rare inflammatory sequela of viral infections that progresses to dilated cardiomyopathy (DCM), heart failure (HF), transplantation, and death in one-third of patients (1–3) and contributes to a substantial number of sudden deaths in young (<40 years) adults (4). Definitive diagnosis of myocarditis is performed by the Dallas Criteria during histopathological examination of endomyocardial biopsy (EMB) (5), however sampling error reduces the sensitivity. Prognosis of myocarditis patients is a critical need with few biomarkers that predict outcomes early in disease (3). Heart-directed autoantibodies (AAbs) have been associated with greater cardiac risk but the mechanisms of AAb-mediated heart injury remain unknown (6–11).

Multiple studies indicate AAbs may be critical in the pathogenesis of myocarditis. The presence of cardiac-directed AAbs was predictive of outcomes in an immunosuppressive therapeutic trial with retrospective analysis of myocarditis patients (12) and purified anti-cardiac myosin (CM) IgG induced myocarditis with inflammatory cell infiltrate as well as DCM when passively transferred into BALB/c mice or Lewis rats (13, 14). Although several different specificities of AAbs have been described by Maisch (15), Kaya (16), and others (17–19), none are currently used as biomarkers for diagnosis or prognosis of disease. CM AAbs have been associated with DCM, fibrosis, and HF (2, 20–26), and CM is one of the few heart proteins which can induce myocarditis when administered to susceptible animal models (7, 27–32). A recent study suggests that elevated serum AAbs against the heart, the female sex, fulminant onset, and lower ejection fraction are all predictors of death or transplantation in myocarditis before the introduction of immunosuppressive therapies (33).

Our previous studies of experimental autoimmune myocarditis (EAM) in Lewis rats demonstrated *in vivo* IgG myocardial deposition as a hallmark of myocarditis pathology (14). *In vivo* IgG deposition in heart tissue also followed passive transfer of serum IgG purified from rats immunized with CM. IgG deposition and dilated cardiomyopathy in heart tissues has also been reported in mouse models of CM-induced myocarditis (34), but not linked to poor outcomes or heart function. Mouse models of viral myocarditis (murine cytomegalovirus (MCMV) and Coxsackievirus B3(CVB3)) have also reported CM AAb in the serum during acute and chronic myocarditis as well as cardiac deposition of CM AAb (35, 36). CM AAbs have also been associated with other types of inflammatory heart diseases (37–42) including poor outcomes in type 1 diabetes (43, 44).

CM AAbs were shown by our laboratory to cross-react with βARs, and passive transfer of CM AAbs caused cardiomyocyte apoptosis and DCM in the Lewis rat model of myocarditis, resulting in cAMP-dependent protein kinase A signaling activation in cardiomyocytes (14, 20). CM AAbs in the Lewis rat model were specific for βAR as they did not react with α-adrenergic receptors (αARs) (14). Similarly, Jahns et al. demonstrated pathogenicity of AAbs against the βARs where passive transfer of sera induced dilated cardiomyopathy in a mouse model of EAM (18). Other studies have shown anti-βAR AAbs associated with idiopathic arrhythmias and myocardial sites of focal infiltration and necrosis in human biopsy tissue (45). β-blocker therapy with carvedilol was efficacious in chronic heart failure patients with AAbs to the βAR (46). While approximately 70% of patients with myocarditis recover left ventricular ejection fraction (LVEF), biomarkers to identify patients who will not spontaneously recover their LVEF is an unmet clinical need to inform treatment decisions and prevent progression to DCM, HF, and transplant (47). Although AAbs against CM may be present in patients with cardiomyopathy and experimental animals with myocarditis, the mechanism of pathogenesis and significance of the anti-CM AAbs is unknown. We show herein that CM/βAR AAbs are elevated at clinical presentation in sera of patients who fail to recover LVEF by one year. In our longitudinal myocarditis cohort, CM AAb titers correlated with lowered EF, and a human myocarditis-derived human monoclonal antibody (mAb) with specificity for both CM and βARs induced fibrosis pathways in H9c2 cells. Our evidence suggests CM AAbs potentially have deleterious effects that may lead to poor outcomes including IgG deposition associated with fibrosis in the myocardium. Endomyocardial biopsies of myocarditis patients had diffuse IgG deposition associated with fibrosis. Importantly, we report CM peptide epitopes recognized by myocarditis sera and identified CM peptide epitopes of the non-recovered phenotype which may be investigated as future biomarkers of more severe disease. Sera from the non-recovered phenotype contained significantly more reactivity to CM peptides overall compared to recovered and healthy sera. The CM peptides that were reactive with myocarditis sera, including the unique peptides recognized by the non-recovered sera, had strong amino acid sequence homology with extracellular loops from both β1AR and β2AR. Thus, our study suggests that CM/βAR AAbs contribute to the pathogenesis of human myocarditis and may in future cohorts identify myocarditis patients who will fail to recover normal heart function. The elevated protein kinase A (PKA) activation by myocarditis sera and the human mAb may indirectly play a role in pathogenesis as Th17 cells are reported to express βAR (48–50) and may be affected by autoantibody mediated PKA-βAR dependent activation. The evidence is important in understanding the pathogenesis of myocarditis, and how it translates to inform therapeutic targets as well as diagnostic biomarkers.

## Methods

### Patient cohort and experimental design

Forty-one adults (age 18–89 years, 66% male) with a diagnosis of acute myocarditis/DCM and 32 healthy adult volunteers (age 18–69 years, 59% male) were enrolled in the study. Timing of blood collection, deaths, or dropouts resulted in sample sizes of less than 41 patients in some assays. All myocarditis/DCM patients were enrolled less than 6 months from date of symptom onset, met clinical criteria for myocarditis, had ejection fractions less than 50%, and had no greater than 50% stenosis in any epicardial coronary artery (1). Patients were further characterized by (a) endomyocardial biopsy that met Dallas criteria for myocarditis/borderline myocarditis, (b) cardiac MRI scan that met consensus conference diagnostic criteria for acute myocarditis, or (c) echocardiogram demonstrating LVEF less than 50% with an otherwise unexplained rise in troponin. All subjects had clinical evaluation and blood collection during their first patient visit (baseline), and blood from a subset of patients was collected at 1 to 3 and 6 to 12 months after baseline. Serum was stored at –80°C. Viral myocarditis was not determined in the cohort by PCR or viral culture from peripheral blood or heart biopsies.

### Antibody staining of heart biopsies

Goat anti-human IgG (10 µg/ml), or isotype-control human IgG Ab (10 µg/ml; Sigma-Aldrich) was incubated on de-paraffinized tissue sections overnight at 4{σπ}°{/σπ}C. Biotin-conjugated rat anti-goat IgG Ab (1/500; Jackson ImmunoResearch Laboratories) was incubated on tissues for 30 min. Alkaline phosphatase-conjugated streptavidin (Jackson ImmunoResearch Laboratories) was incubated on tissues at 1 µg/ml for 30 min at room temperature. Ab binding was detected with Fast Red substrate (BioGenex) against a counterstain of Mayer’s hematoxylin (BioGenex). Cell surface-bound Abs were detected with specific biotin-conjugated secondary Ab (1/500; Sigma- Aldrich).

### Trichrome staining of heart biopsies

Tissue sections were stained in our laboratory using Masson’s Trichrome Method. Briefly, tissues were de-paraffinized and rehydrated through alcohol gradients, washed in distilled water, then re-fixed in Bouin’s solution [saturated picric acid, formaldehyde (37-40%), glacial acetic acid] for 1 hour at 56°C, or overnight at room temperature when formalin fixed. The tissues were cooled and washed in running water to remove yellow color, then rinsed in distilled water. Sections were then stained in Wiegert’s iron hematoxylin solution [stock solution A: hematoxylin and 95% alcohol; stock solution B: 29% ferric chloride in water, distilled water, concentrated hydrochloric acid; working solution - equal parts of stock solutions A and B for 10 minutes, washed in warm running water for 10 minutes, and rinsed in distilled water. The tissues were then stained in Biebrich scarlet-acid fuchsin solution (Biebrich scarlet, 1% aqueous, acid fuchsin, 1% aqueous, and glacial acetic acid) for 10 minutes, then rinsed in distilled water. Tissues were differentiated in phosphomolybdic-phosphotungstic acid solution (5% phosphomolybdic acid, 5% phosphotungstic acid) for 10 minutes. The solution was discarded, and tissue sections were stained with aniline blue solution (aniline blue, glacial acetic acid, distilled water) for 5 minutes. Sections were briefly rinsed in distilled water, placed in 1% glacial acetic acid solution for 3 minutes, washed in distilled water, then dehydrated in 95% ethyl alcohol, absolute ethyl alcohol, and cleared in xylene, two changes each. Sections were mounted with Permount. In trichrome-stained tissue sections, nuclei stain black and collagen stains blue. Cytoplasm, keratin, muscle fibers and intercellular fibers stain red.

### ELISA

Antigen human CM was prepared as previously described (38). Peptides of the S2- fragment were diluted at 10 µg/mL in carbonate/bicarbonate buffer and 50 µL/well placed into each well of a 96-well ELISA plate. The plates were covered in plastic wrap and incubated overnight at 4°C. The plates were washed 5X with PBS/0.5% Tween. The plates were blocked by dispensing 100 µL/well of 1% BSA/PBS and incubating for one hour at 37°C. The plates were washed as described above and 50 µL/well of diluted (1:100) sera placed on the plate in duplicate wells. The plates were covered in plastic wrap and placed overnight at 4°C. The following day, the plates were washed. Secondary antibody conjugated to alkaline phosphatase was diluted to 1:500 in 1% BSA/PBS and 50 µL/well added and plates left to incubate for one hour at 37°C. The plates were then washed, and substrate solution added at 50 µL/well. The optical density was read at an absorbance of 405 nm after 15, 30, 60, and 90 minutes. Results shown in graphs are from the 60 min timepoint.

### S2 peptide synthesis

Peptides spanning the S2 fragment (hinge region) of the human cardiac myosin molecule were synthesized and purified by Genemed Synthesis, Inc. (San Francisco, CA). These S2 fragment peptides were synthesized as 25-mers with 11-amino acid overlap with the following amino acid sequences (20): S2-1 SAEREKEMASMKEEFTRLKEALEKS, S2-2 FTRLKEALEKSEARRKELEEKMVSL, S2-3 RKELEEKMVSLLQEKNDLQLQVQAE, S2-4 KNDLQLQVQAEQDNLADAEERCDQL, S2-5 LADAEER CDQLIKNKIQLEAKVKEM, S2-6 KIQLEAKVKEMNERLEDEEEMNAEL, S2-7 LEDEEEMN AELTAKKRKLEDECSEL, 2-8 KRKLEDECSELKRDIDDLELTLAKV, S2-9 IDDL ELTLAKVE KEKHATENKVKNL, S2-10 KHATENKVKNLTEEMAG LDEIIAKL, S2-11 MAGLDE IIAKLTKEKKALQEAHQQA, S2- 12 KKALQ EAHQQ ALDDLQAEEDKVNTL, S2-13 LQAEEDKVNTLTKAKVKLEQ QVDDL, S2-14 KVK LEQQ VDDLEGSLEQEKKVRMDL, S2-15 LEQEKKVRMDLER AKRKLEGDL KLT, S2-16 KRKLEGDLKLTQESIMDLENDKQQL, S2-17 IMDLENDKQ QLDERLKKKDFELNAL, S2-18 LKKKDFELNALNARIEDEQALGSQL, S2-19 IEDEQALGSQLQ KKLKELQARIEEL, S2-20 LKELQARIEELEEELESERTARAKV, S2-21 LESERTAR AKVEKLRSDLSR ELEEI, S2-22 RSDLSRELEEISERLEEAGGATSVQ, S2-23 LEEA GGATSVQIE MNKKREAEFQKM, S2-24 NKKREAEFQKMRR DLEEATLQ HEAT, S2-25 LEEATLQHEATAAALRKKHADSVAE, S2-26 LRKKHADSVAEL GEQIDNL QRVKQK, S2-27 QIDNLQRVKQKLEKEKSEFKLELDD, S2-28 EKSEFKLELD DVTSNMEQIIKAKAN, S2-29 NMEQIIKAKANLEKMCRTLEDQMNE, S2-30 MCRTLED QMN EHRSKAEETQRSVND, S2-31 KAEETQRSVNDLTSQRAKLQTENGE, S2-32 ETQR SVNDLTSQRAKLQTENGELSR.

### Protein kinase A assay

H9c2 rat cardiac myoblast cell line (ATCC) (1 x 10^7^) were plated in T75 cell culture flasks overnight at 37^0^ at 5% CO2 for use in the protein kinase A assay. H9c2 has been demonstrated to express the βAR receptor (14). Serum samples were incubated with the H9c2 cells at a 1:100 dilution in a final volume of 15 mL of serum-free medium for 1 hour before reactions were stopped by an ice-cold PBS (10 mL) rinse. The cells were mechanically dislodged from the flasks, centrifuged, and solubilized in 0.3 mL of protein extraction buffer before homogenization. Each pellet was homogenized with chilled homogenizer for 30 seconds, on ice. PKA activation in H9c2 cells was measured using SignaTECT cAMP-dependent protein kinase assay system (Promega) according to the manufacturer’s instruction. The specific activity of the enzyme in picomoles per minute per microgram for each sample was calculated and results were presented as a percentage above the basal PKA rate (20, 51).

### Human B cell hybridoma production

Human hybridoma production was performed by routine methods in our laboratory for creating and maintaining hybridomas and producing human monoclonal antibodies (mAbs) (42, 52–54). Peripheral blood was obtained from a 54-year-old female diagnosed with DCM. Peripheral blood mononuclear cells were separated from whole blood by Histopaque-1077 (Sigma) and stimulated for 1 week with pokeweed mitogen (2 µg/mL) in Iscove’s Modified Dulbecco’s Medium (IMDM) containing 10% human AB serum, 50 µg/mL gentamycin, and 50 IU/mL penicillin/50 µg/mL streptomycin. Cells were washed three times in IMDM (serum-free) and fused with HMMA2.11TG/0 cells (human/mouse myeloma cell line). The HMMA cell lines have been described by their inventor Dr. M. Posner (55). HMMA 2.11TG/O was the fusion partner with the human lymphocytes purified by ficoll-hypaque gradient from the peripheral blood of the myocarditis patient. HMMA cells were added to the lymphocyte suspension in a 1:1 ratio and centrifuged together at 1000 rpm for 7 minutes. Three milliliters of 35% polyethylene glycol (PEG-1000) in IMDM pH 7.8 were dripped down the side of the tube. The cell mixture was gently agitated and centrifuged at 900 rpm for 2 minutes. The cells were then “rested” for 6 minutes at 30^0^C. Forty milliliters of IMDM containing 10% fetal bovine serum (FBS) was gently dripped down the side of the tube. The tube was centrifuged at 900 rpm for 8 minutes and the cell pellet was re-suspended in IMDM containing 10% (FBS), gentamycin, and penicillin streptomycin. The cells were plated in 24-well tissue culture plates at 1x10^6^ cells/mL and 0.5 mL/well was placed into each well and incubated overnight. The next day, 0.5mL HAT (hypoxanthine, aminopterin, and thymidine) medium was added to each well. On day 3 after the fusion, 0.5 mL fresh HAT medium was added to each well. On days 5 and 10, 1 mL fresh HAT medium was added to each well. Starting on day 12, the medium was switched to HT (hypoxanthine and thymidine) and 1 mL fresh media was added every 3-5 days as hybridoma growth was monitored. Culture fluid from wells positive for hybridoma growth was screened in the ELISA against antigens CM and β1AR. Cloning of hybridomas was achieved by limiting dilution and rescreening with antigens and was performed two times. Established clones were maintained in IMDM containing 20% (FBS) eventually reduced to 10% FBS for final fluid collection for study. Assays were compared to the media control containing FBS. Positive clones were identified for immunoglobulin isotype and concentration using standard curves and isotype-specific secondary antibodies.

### Monoclonal antibody 2C.4

Human hybridoma cell lines were selected by supernatant reactivity to βAR and PKA activity by ELISA, cloned twice, stored, and maintained as described above. The human mAb used in this study included 2C.4 which was stored as culture fluid at 4°C. Clone 2C.4 was screened against human cardiac myosin and the βAR enriched membranes (Perkin Elmer) by ELISA, and by PKA activity. Our process resulted in several clones with strong reactivity to β1AR and β2AR (**Supplementary Figure 1**). We selected clone 2C.4 because it was highly cross- reactive with human cardiac myosin, had the highest reactivity to both βAR1 and 2, and activated PKA in the H9c2 heart cell line.

### Gene expression in treated and untreated H9c2 (ATCC) heart cell line

The rat cardiac myoblast/primary heart cell line H9c2 was described by ATCC as: “a subclone of the original clonal cell line derived from embryonic BD1X rat heart tissue that exhibits many of the properties of skeletal muscle. This cell line is recommended for cardiovascular disease research (56).” The H9c2 cell line was treated in triplicate for 30 minutes with 1:100 dilution of sera from myocarditis/DCM patients (n=2) or healthy subjects (n=2), left untreated, or treated with 0.882 ug/mL of mAb 2C.4. Following treatment, RNA was isolated from individual wells using TRIzol. RNA concentrations were quantified using a Qubit fluorometer, and mRNA libraries were generated using 500 ng of each RNA sample and Illumina’s TruSeq Stranded mRNA Library Prep Kit. Bulk PBMC RNA-seq was performed on the Illumina HiSeq 3000 (Illumina Inc., San Diego, CA) in the OMRF’s Clinical Genomics Center following Center procedures. Samples were sequenced using 75bp paired-end reads with 10 samples per lane, which yielded ~30 million reads per sample. Post-sequence reads were quality filtered and trimmed for Illumina adapters using Trimmomatic v0.35 (57). Resulting reads were pseudo-aligned to coding regions of the Rnor_6.0 genome (release 101) using Kallisto v0.44.0 (58) with the following options: bias enabled, 50 bootstraps. Expression values for transcripts were measured in transcripts per million reads sequenced (TPM). Gene annotation was performed via the R package biomaRt (59). To perform differential expression (DE) analyses, expression values were summarized at the gene level to transcript-length adjusted, library-size scaled counts per million (CPM) with the R package tximport (60). DE was calculated using the empirical Bayes approach implemented in the R package DESeq2 (61). Significantly DE genes had an absolute value log2 fold change ≥ 1.

### Pathway analyses

To characterize pathways, we utilized a commercial software package, Ingenuity Pathway Analysis (IPA) to intersect DE genes with known biological functions or pathways, maintained in the IPA database, a collection of nearly 5 million experimental findings manually curated from either literature or third-party databases. Detailed information can be found at http://qiagen.force.com/KnowledgeBase/. Enriched pathways are identified via the Canonical Pathways function. The significance of the overlapping genes from DE genes and genes within the enriched pathway is reported as a p-value, calculated by the right-tailed Fisher’s Exact Test. In addition to a p-value, Canonical Pathways reports the percent overlap (given as a ratio) and a z- score, a measure of activation or inhibition of a pathway based on the observed and predicted magnitude and direction of fold changes in overlapping genes. Calculations of z scores have been described in detail (62). Here, a pathway was defined as enriched if its IPA Z score was above 1 or below -1 and had a significant p-value (<0.05).

### Confirmation of RNAseq by qPCR

Eight genes were selected from our RNA sequencing result for confirmation by qPCR via an independent experiment with parallel design to our RNA sequencing experiment. As described above, H9c2 rat heart cells were incubated with patient (n=6) and healthy subject (n=4) sera (1:100) for one hour in medium and washed with ice-cold PBS. Cells were immediately lysed with 1ml TRIzol (Invitrogen) and stored at -80°C. RNA was isolated by chloroform extraction according to manufacturer’s (Invitrogen) instructions. RNA cleanup was performed by passing isolated RNA through silica columns (RNA Clean & Concentrator kit, Zymo Research.). cDNA was reverse transcribed from 0.35 ug RNA with OmniScript Reverse Transcriptase (Qiagen), diluted 1:10, and used directly in 25 ul SYBR Green (QuantiTect SYBR Green qPCR Kits, Qiagen) qPCR reactions at a final dilution of 1:125. Pre-designed and validated primers (KiCqStart SYBR Green Primers, Millipore Sigma) were used at a final concentration of 0.5 µM. 40 cycles of qPCR were carried out on ABI 7500 RT-qPCR system, and CT threshold set in the linear range of amplification, and a single amplicon was verified by a single product present in the dissociation curve. Non-specific amplification was controlled through control reactions lacking template, and with no-RT controls. Log2 fold changes were calculated using the 2^-ΔΔCt^ method (63), and statistical significance between groups was determined using a two-tailed unpaired t-test.

### Statistical analyses

For variables including anti-CM titer and PKA activation, when the normality of the distribution was rejected, a nonparametric Kruskal-Wallis test was used for comparisons of medians among 3 or more groups, followed by *post-hoc* testing using unpaired Mann-Whitney *U* tests with a Bonferroni-adjusted alpha level. One-way ANOVA with a Tukey’s *post-hoc* multiple comparison test was used to compare more than 3 means when normality was satisfied. Correlation between CM titer and echo LVEF was determined by using the Spearman’s rank correlation test. A 2-sided p-value less than or equal to 0.05 was considered statistically significant, unless otherwise specified, where a more stringent, or conservative, alpha level less than 0.05 was used to define significance due to multiple comparisons. Bar graphs reflect the mean ± SEM, while the dot plots include a horizontal line drawn at the median value for each subgroup.

### Reporting sex and gender based analyses

Although we evaluated both sexes in our study, more males (n=27) than females (n=14) are found to have myocarditis and the number of the non-recovered patients was small (n=6) with 3 males and 3 females in the non-recovered cases studied. Our previous study of this cohort did compare males and females and is published (6). Males were dominant for biomarkers of non-recovery such as Il-17A and IL-6, and in another study, elevated ST-2 dominated in males with myocarditis. Here the comparison of CM Ab titers in males vs females was 25 males to 12 females with no statistical differences observed.

### Study approval

The use of subject blood and data required for our studies was approved by Institutional Review Boards at OUHSC and Mayo Clinic. Written informed consent was obtained from participants before study initiation according to the Declaration of Helsinki with regard to scientific use. Peripheral blood was obtained from healthy subjects who were laboratory volunteers at OUHSC or were healthy donors from the Oklahoma Blood Institute in Oklahoma City, Oklahoma.

### Data availability

Sequencing data can be accessed through NIH National Center for Biotechnology Information under BioProject accession PRJNA1025010.

## Results

### Non-recovered myocarditis patients at baseline show significantly higher cardiac myosin and cardiac myosin peptide autoantibodies than those who recover

**Figure 1:**
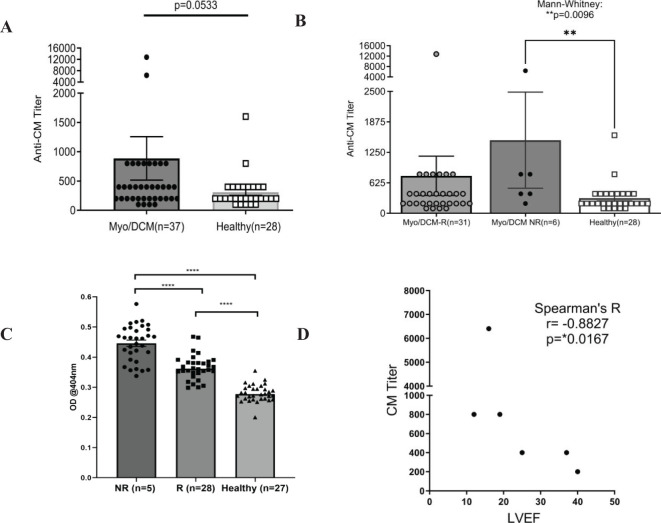
Baseline autoantibodies to CM peptides are significantly elevated in non-recovered (NR) myocarditis patients compared with recovered (R) patients. Sera taken at initial clinical presentation from recovered and non-recovered myocarditis were reacted with whole CM molecule or 32 overlapping peptides of CM in ELISA. CM titers strongly trended towards significance when all patients and healthy subjects were compared **(A)** Non-recovered patients had significantly elevated titers to CM compared to healthy controls **(B)**. In **(C)**, non-recovered (n=5) myocarditis patients have significantly elevated autoantibodies to CM peptides when compared with recovered (n=28) patients and healthy subjects (n=27). In **(C)**, each datapoint is the average serum IgG immunoreactivity (OD @ 1:100 serum dilution) with all 32 S2 CM fragment peptides for each individual (i.e. each black dot is the average from all patients for one peptide). Serum IgG titers to CM correlated with lower ejection fraction in non-recovered patients, but not in recovered patients [**(D)**,s **Supplementary Figure 2**] NR, Non-recovered; R, Recovered.

Elevated CM AAbs may suggest non-recovery of LVEF in myocarditis and dilated cardiomyopathy, and we tested this hypothesis in a longitudinal study of myocarditis/DCM patients where recovery was defined as reaching >50% LVEF by one year after onset. Both recovered and non-recovered myocarditis/DCM patients were analyzed for IgG reactivity to human CM, and to 32 overlapping synthetic peptides of the S2 hinge-region fragment of CM (CM peptides). Sera was collected at initial clinical presentation at baseline, no more than 6 months past onset. Sera were tested by ELISA for IgG reactivity to CM and CM peptides. In all of the myocarditis patients in this study, elevated titers of serum CM AAbs trended towards significance (p=0.0533) in baseline samples compared to healthy subjects (**Figure 1A**). However, highly significant elevated CM AAb titers were found in our cohort of non-recovered patients (n=6) (**Figure 1B**, p=0.0096) compared to normal healthy subjects. Elevated CM AAbs in the non-recovered group suggest that CM AAbs may be a biomarker of disease progression similar to AAbs in other autoimmune diseases (64). Other timepoints were not significant. We also analyzed AAb reactivity to peptides from the S2 hinge region of CM, and we hypothesized that reactivity to all peptides may be more important than any single one. We averaged all patient IgG reactivity for each given peptide and compared the means of all peptides measured in the recovered group with the non-recovered group. Importantly, baseline sera IgG reactivity to S2 peptides was significantly elevated in non-recovered myocarditis/DCM patients when compared with recovered patients (**Figure 1C**, p<0.0001), suggesting that IgG reactivity to CM peptides may be important in determining disease outcomes.

To study the relationship of CM AAb reactivity to outcomes, we correlated LVEF with AAb titers in our patient cohort. LVEF did not significantly correlate with CM AAb titers in patients who recovered LVEF (**Supplementary Figure 2**, r=-0.06274, p=0.7559), however, the subset of myocarditis patients (n=6) who did not recover LVEF (non-recovered) were found to have high CM AAb titers which correlated with low LVEF (**Figure 1D**, r= -0.8827, p=0.0167). Removal of one apparent outlier (patient with AAb titer 6400) resulted in an even stronger correlation (**Supplementary Figure 3**, r = -0.9487, p= 0.0333). CM AAbs negatively correlated with LVEF in non-recovered patients, suggesting a functional role of CM AAbs in progression of myocarditis.

**Figure 2:**
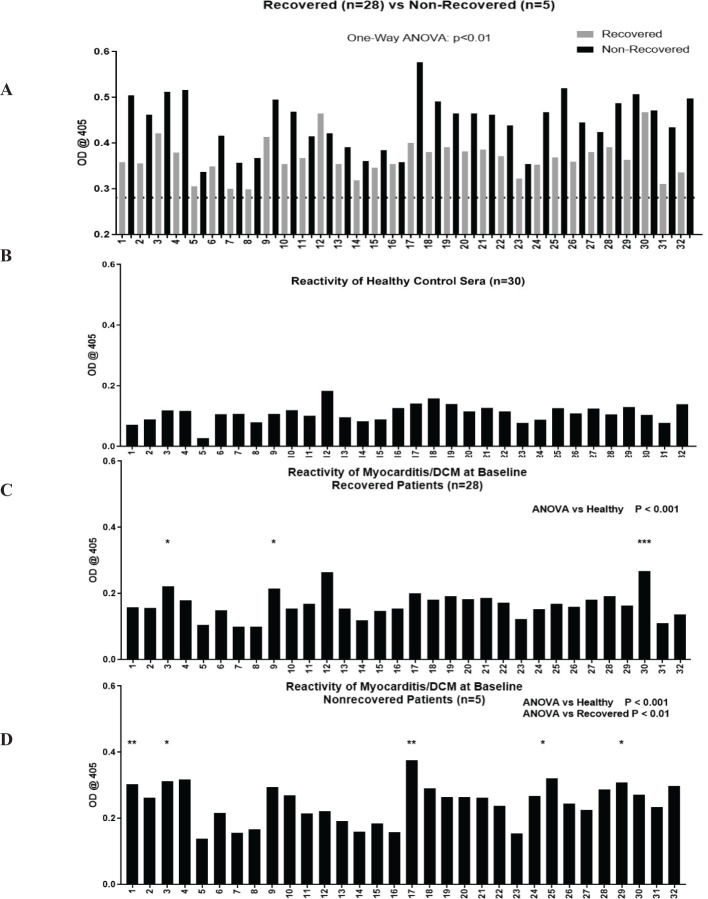
Autoantibodies to CM peptides are elevated in non-recovered patients and AAbs recognize unique CM peptide epitopes in non-recovered patients. Overlapping peptides synthesized from the S2 region of CM were tested by ELISA for reactivity with serum IgG in healthy (n=30, panel **B**), recovered (n=28, panel **C**), and non-recovered patients (n=5, panel **D**). Recovered and non-recovered are shown in the top panel **(A)**, dotted black line indicates mean healthy subject (n=30) serum IgG antibody reactivity at mean OD of 0.2809 ± 0.03. OD values normalized to healthy are plotted in **(B-D)**. One-way ANOVA showed groupwise differences between healthy compared with recovered (panel **C**, p <0.0001) and with non-recovered (panel **D**, p <0.001). Post-hoc analysis showed that in recovered patients compared with healthy control serum IgG, peptides S2-3 (p <0.01), S2-9 (p <0.01), and S2-30 (p <0.0001) reached significance after multiple test correction. IgG AAbs to CM peptides specific to non-recovered patient sera were S2-1 (p <0.001), S2-3 (p <0.01), S2-17 (p <0.001), S2-25 and S2-29 (both p <0.01) reached significance above healthy subjects. ANOVA showed groupwise differences between recovered and non-recovered patients (panel **D**, p <0.01). The asterisks refer to the p value: * p <0.01, ** p < 0.001, *** p < 0.0001.

We sought to identify unique CM peptide epitopes of non-recovery in myocarditis patients. OD measurements for all individual S2 hinge region peptides are shown in **Figure 2A**, where grey bars show the average OD for all recovered patients, and black bars correspond to non-recovered patients. The S2 hinge region was chosen in our studies because it was demonstrated to be highly immunogenic in previous studies (20), likely due to its proteolytic sensitivity (65). The dashed line on **Figure 2A** indicates the mean healthy subject IgG reactivity (optical density at 0.2809 ± 0.03) (**Figure 2A**) and reactivity of healthy control sera for each peptide is shown in **Figure 2B**. Groupwise analysis of all 32 peptides showed significant differences between healthy compared with both recovered and non-recovered patient groups (One-way ANOVA, both p < 0.0001), and *post-hoc* analysis revealed several individual peptides that reached significance after multiple test correction (**Figures 2C, D**). *Post-hoc* analysis showed that in recovered patients compared with healthy control serum IgG, peptides S2-3 (p <0.01), S2-9 (p <0.01), and S2-30 (p <0.0001) reached significance after multiple test correction (**Figure 2C**). IgG AAbs to CM peptides specific to non-recovered patient sera were S2-1 (p <0.001), S2-3 (p <0.01), S2-17 (p <0.001), S2-25 and S2-29 (both p <0.01) reached significance above healthy subjects (**Figure 2D**). Further, recovered patients compared with non-recovered was also significant (One-way ANOVA p<0.01). Therefore, significantly elevated IgG specific for S2 CM peptides at the time of clinical presentation may suggest that the epitopes recognized in the CM S2 peptides may be important in identifying the non-recovered phenotype in human myocarditis/DCM. (**Supplementary Tables 1A, B** show the locations of the CM epitopes as recognized by AAbs in myocarditis sera).

### Myocarditis/DCM sera IgG activates protein kinase A significantly higher than healthy sera

**Figure 3:**
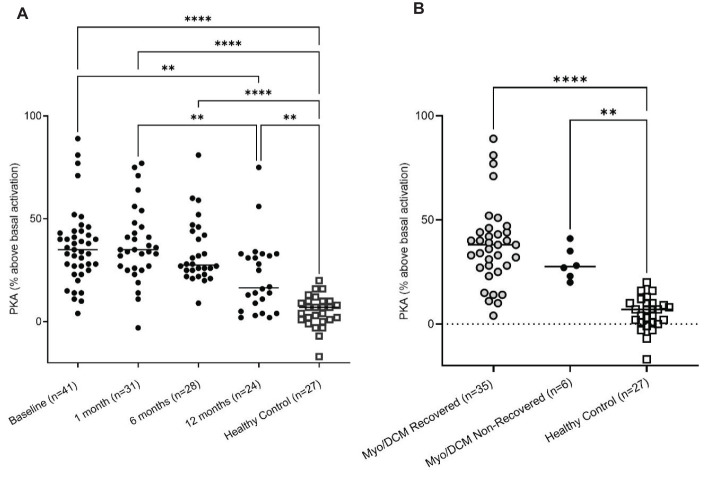
Myocarditis/DCM sera signal PKA H9c2 cells treated with myocarditis/DCM sera (1:100 dilution). **(A)** Levels of PKA signaling in H9c2 cells at various timepoints are compared to healthy subject levels. **(B)** PKA signaling is significantly elevated in both recovery and non-recovery at the baseline time point. Recovered is shown in One-way ANOVA compared with healthy sera with Tukey’s multiple comparison test ****p<0.0001, non-recovered vs healthy sera: **p=0.0023. Although myocarditis sera strongly signal PKA, there is no difference in the recovered vs non-recovered.

We previously demonstrated that AAbs specific for cardiac myosin cross-react with βARs and activate PKA (14, 20). Activation of cyclic AMP-dependent PKA is a key step in cardiomyocyte contraction, and overstimulation of this pathway may lead to cardiomyocyte apoptosis and contribute to dilated cardiomyopathy (14). CM AAbs bind directly to both β1AR and β2AR, and signaling through PKA can be inhibited pharmacologically by βAR antagonists and by pre-incubation of AAbs with soluble β1AR, β2AR, and human cardiac myosin (14, 20). In **Figure 3** we demonstrate that myocarditis/DCM sera activate PKA signaling in a rat heart cell line using a P^32^ detection assay. Myocarditis/DCM sera at each time point (baseline, 1, 6, and 12 months) activated PKA significantly higher than healthy sera (**Figure 3A**), however no significant differences in PKA activation between recovered and non-recovered patients were observed (**Figure 3B**). PKA activation was significantly (p=0.0061) higher at the myocarditis baseline blood draw compared to the 12-month blood draw, perhaps related to recovery in most patients by 12 months. In a small myocarditis patient sample set we verified that, as in our previously published work (14, 20), PKA signaling in our current cohort was indeed due to IgG mediated signaling (n=3, **Supplementary Figure 4**). Incubation of myocarditis sera with propranolol diminished PKA activation, and anti-IgG-coated beads diminished PKA activity in a similar magnitude while BSA-coated beads demonstrated no effect (**Supplementary Figure 4**). In our previous studies, PKA signaling was blocked >80% by co-incubation of CM AAbs with antigens CM, β1AR, and β2AR, demonstrating cross-reactivity of the CM AAbs with βAR (20). In context with previously known properties of CM AAbs, our present results suggest PKA signaling induced by myocarditis/DCM sera reflects βAR activation by AAbs specific for both CM and the βAR.

### Human myocarditis derived mAb 2C.4 induces fibrosis pathways in H9c2 cells

**Figure 4:**
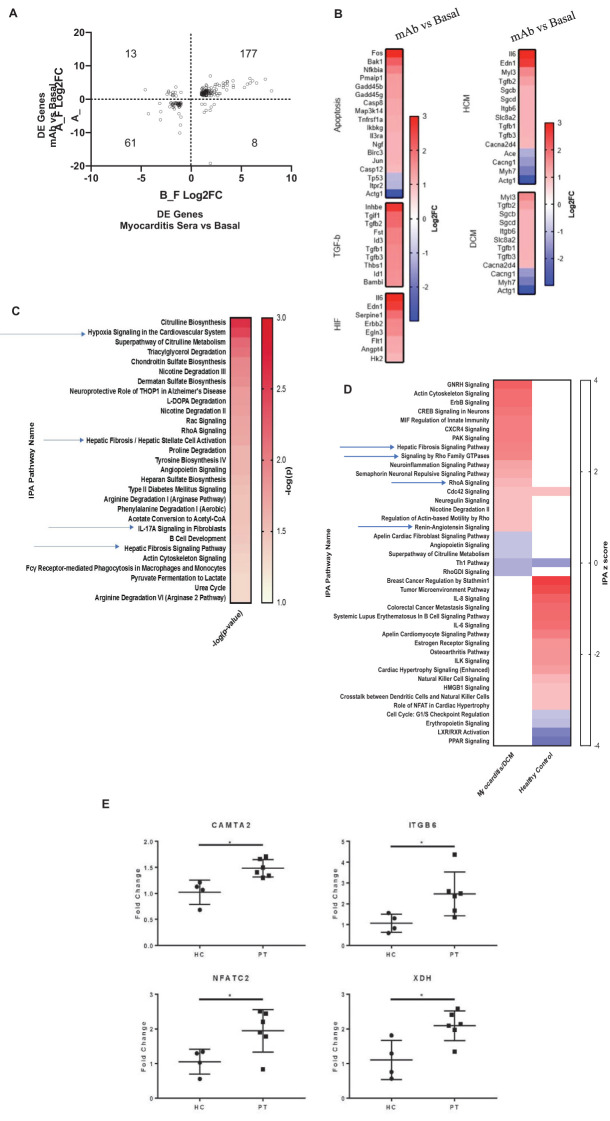
Myocarditis mAb 2C.4 and sera promote a fibrotic transcriptome in H9c2 cells. **(A)** DE genes in mAb/sera vs. basal after treatments of H9c2 cells. Log2FC values DE genes show high concordance (same direction) in mAb/sera treated cells: DE genes in upper-right (177) vs lower left (61) quadrants. **(B)** KEGG Analysis of DE genes: mAb compared to basal treated H9C2 cells. **(C)** IPA of DE genes in myocarditis vs healthy (n=2) serum treated cells shows enriched fibrosis/hypoxia/apoptosis pathways. Red positive z score; blue negative z score indicates DE direction. **(D)** IPA of DE fibrosis related genes elevated in myocarditis serum treated cells (upper left) but not elevated in cells after treatment with healthy serum (lower right) p <0.05. **(E)** By qPCR, fibrosis/heart failure genes were upregulated in cells after treatment with myocarditis (PT, (n=6)) serum (CAMTA2, ITGB6, NFATC2, and XDH) vs healthy control (HC) sera (HC, n=4) treated H9C2 cells. Data analyzed with ΔΔCT method; β-actin reference gene. A gene was considered DE if it demonstrated Log_2_-fold change > or < 1 (red and blue, respectively).

In myocarditis, serum AAbs may cause potentially pathogenic transcriptional changes in cardiomyocytes. H9c2 cells were treated with myocarditis patient sera and also treated with human myocarditis derived mAb 2C.4 and compared with untreated cells to identify differentially expressed genes. Compared with untreated H9c2 cells, mAb 2C.4 induced 1296 DE genes, and patient sera induced 472 DE genes. Over half of these DE genes from patient sera (259/472, 55%) were also differentially expressed by mAb 2C.4 treatment. Nearly all (238/259) of these were concordant in sign (i.e. positive in both analyses, or negative in both analyses) (**Figure 4A**). Ingenuity pathway analysis of myocarditis sera-induced pathways showed upregulation and activation of fibrosis pathways (**Figures 4B–D**). We also performed an independent RT-qPCR experiment with parallel design, where H9c2 cells were treated with serum from 6 different myocarditis patients and 4 healthy subjects, analyzed with the ΔΔCT method (63) with β-actin as the reference control gene. Myocarditis sera induced CAMTA2, ITGB6, NFATC2, and XDH gene expression significantly (p <0.05) above healthy sera (**Figure 4E**). These four genes are related to heart failure and cardiovascular events and are discussed below.

**Figure 5:**
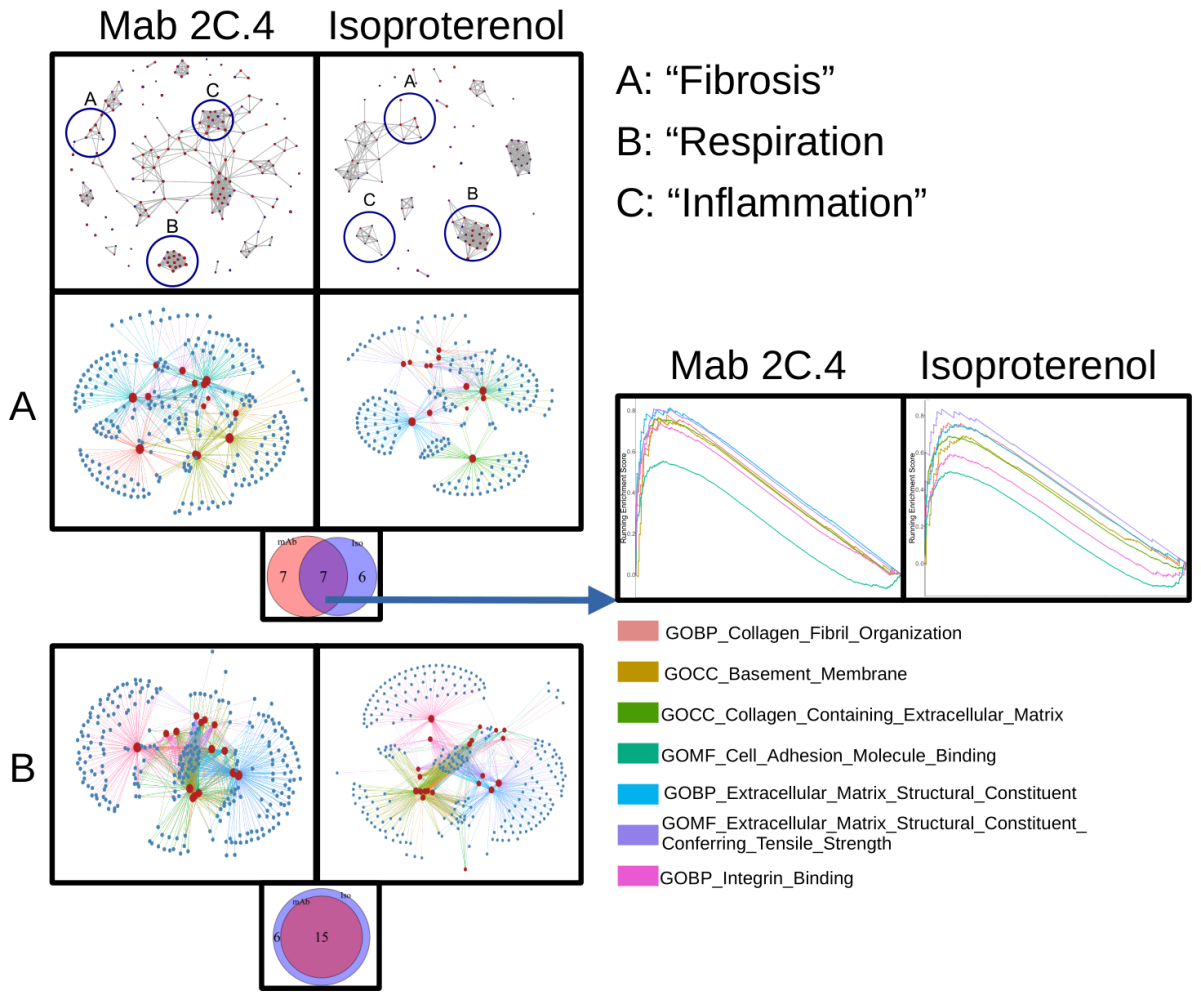
Myocarditis-derived mAb 2C.4 induces fibrosis-related pathways in heart cells. Heart (H9c2) cells treated with human mAb 2C.4 or β-adrenergic agonist isoproterenol (ISO) underwent RNA sequencing, then pathway analysis (GSEA). Functional units (FU) visualized with emapplot() from enrichplot (top panel, left 2C.4, right ISO) identified fibrosis, respiration, and inflammation FUs from significantly upregulated pathways (Circled and labeled "A", "B", and "C". Redundant pathways were ruled out with leading edge analyses (Panels **A** and **B**, correspond to FU **A**: fibrosis and **B**: respiration). No shared pathways were observed in FU in C containing inflammatory pathways, therefore, **(C)** was not considered for further analysis. Thus, C is not shown below separately. **(A, B)** are shown separately to compare the shared fibrosis and respiration pathways in the H9c2 cells. Similarities between responses to mAb 2C4 vs isoproterenol treatment of the H9c2 cells are compared. **(A)** Unique and shared pathways between ISO and 2C.4 are shown in Venn-diagram (inset) and enrichment scores for shared fibrosis pathways compared between conditions (Lower Panel **A**: arrow). GSEA plots (Far Right Panels following arrow) for seven shared fibrosis pathways reveal similar mAb 2C.4 and ISO pathways identified in H9c2 cells after treatment with the serum or the human mAb suggesting shared fibrosis signatures. (Lower Panel **B**) Myocarditis-derived mAb2C.4 induced many respiration pathways in H9c2 cells similar to ISO treatment. Isoproterenol was used as a positive control to compare to the human mAb response in H9C2 cells.

Isoproterenol, a strong βAR agonist, was used as a positive control to compare isoproterenol response to the human mAb 2C4 response in H9C2 cells. Signaling pathways of βAR induced by isoproterenol are well-established and were therefore an appropriate comparison for transcriptomic response of human CM mAb 2C4. To this end, we performed RNA sequencing on mAb 2C.4-treated and isoproterenol-treated (ISO, synthetic βAR agonist) H9c2 cells, and used gene set enrichment analysis (GSEA) (66, 67) to analyze differentially expressed genes (DESeq2). The visualization suite R package enrichplot (68) was utilized to aid in interpretation of the results (**Figure 5**). From the entire list of enriched pathways, we used the function emapplot() to visualize all significantly enriched pathways with a positive enrichment score, and to cluster pathways into functional units (FU) (69) (**Figure 5**, top panel). We identified three pathway clusters, one related to fibrosis, the second involving mitochondrial respiration, and the third related to inflammation (**Figure 5**, top panel, clusters A, B, and C respectively). Because βAR stimulation is known to cause inflammatory changes (70), we compared inflammatory pathway changes between both treatment conditions. However, there were no overlapping inflammatory pathways between treatment conditions, and these pathways were therefore not investigated further. The genes within the leading edge of clusters A and B were visualized using the function cnetplot() from the R package enrichplot (68) (**Figures 5A, B**) to determine whether pathways are independently enriched, or merely represent child-parent relationships. **Figures 5A, B** show that most pathways are enriched from unique sets of genes, with a few redundant (child-parent) pathways It is well established that β-adrenergic stimulation causes an increase in respiration (71–75) and mAb 2C.4 was selected on the basis of βAR binding/PKA signaling, therefore, we hypothesized that we would observe an increase in respiration related pathways similar to ISO treatment. As expected, and according to our hypothesis, all respiration-related pathways induced by ISO were also induced by mAb 2C.4, confirming that 2C.4 induces respiration, and validates our analytical approach (**Figure 5B**). We then compared pathways related to fibrosis between 2C.4 and ISO treatment (**Figure 5A**), and found that like respiration pathways, mAb 2C.4 also directly induces many of the same fibrosis pathways induced by ISO in H9c2 cells. We interrogated the GSEA plots of the seven overlapping pathways (**Figure 5A**, Venn-Diagram) to assess the maximum enrichment score (ESmax), and found that ESmax for fibrosis pathways induced by human mAb 2C.4 were at similar or greater magnitude than that of ISO. We also used the ranked gene list in this analysis as input to STRINGdb for a parallel analysis of enriched pathways. Both treatment conditions showed significant enrichment of fibrosis-related pathways. This analysis yielded a similar result, demonstrating that mAb 2C.4 treatment enriched fibrosis and respiration/adrenergic pathways similarly to ISO treatment. (**Supplementary Figures 5A–D**, **Supplementary Tables 2A–D**, and **Supplementary Tables 3A, B**). Overall, our data shows that the myocarditis-derived cross-reactive CM/βAR mAb 2C.4 induces a transcriptional response consistent with a fibrosis signature and suggests that CM/βAR AAbs in myocarditis and progressive heart failure may contribute to cardiac fibrosis.

### CM IgG AAbs were detected in biopsies from patients with myocarditis

Previously, we demonstrated in EAM that CM AAbs targeted the heart cell surface, and the heart as evidenced by *in vivo* IgG myocardial deposition and AAb passive transfer of DCM (14). To assess AAb binding to cardiomyocytes in human tissue, we de-paraffinized myocarditis biopsy sections and incubated them with anti-human IgG or conjugate control antibodies. Biopsies from myocarditis patient (n=5) sections showed diffuse IgG deposition in the myocardium, while conjugate controls (n=5) and representative healthy subject myocardium did not show IgG deposition within normal heart tissue (**Supplementary Figure 6**). IgG deposition detected in this assay measured endogenous patient IgG bound in myocardium.

## Discussion

The role of humoral immunity in the pathogenesis of autoimmune heart disease has not been established. Although immunization with CM leads to myocarditis with Th17 cellular infiltration in the heart, the mechanisms by which CM Abs may adversely affect the heart have not been determined in humans. Our novel findings show that anti-CM autoantibodies in experimental autoimmune myocarditis cross-react with the βAR and induce signal transduction in heart cells. Our data support the hypothesis that anti-CM autoantibody in myocarditis may have pathophysiological effects on heart cells due to AAb cross-reactivity with the beta adrenergic receptors. Therefore, molecular mimicry and cross-reactivity of cardiac autoantibodies may be an important mechanism by which AAbs may contribute to heart disease. AAbs that target the heart in myocarditis have been previously described, yet no human studies have demonstrated a connection between CM and βAR AAbs and outcomes in the non-recovered phenotype of myocarditis/DCM patients. Several findings in our study support a link between these cross-reactive AAbs and outcomes of myocarditis/DCM.

CM AAbs may cause the non-recovered phenotype, and/or may be a biomarker of risk of non-recovery, thereby aiding in prognosis at an early stage of disease. In this regard, we found a direct correlation between elevated CM AAb titers and lowered EF in myocarditis/DCM in the non-recovered phenotype and found CM AAb reactivity to unique peptide epitopes in the S2 hinge region of CM (**Supplementary Tables 1A–C**). AAbs to these peptide epitopes were significantly elevated in the group of myocarditis patients that failed to recover LVEF and developed progressive heart disease.

CM AAbs may be pathogenic due to their signaling of the βAR on heart cells. Thus, CM AAbs may be functional biomarkers that induce gene expression consistent with known features of pathogenesis, namely fibrosis, where the AAbs might act like isoproterenol signaling the βAR and lead to fibrosis of the heart (76). The link of the CM AAbs with effects of βAR signaling and poor outcomes has not been reported together in the literature or clearly connected with fibrosis in human disease. Further, in our study, endomyocardial biopsies from myocarditis patients with positive IgG deposits frequently demonstrated a high degree of fibrosis (**Supplementary Figure 6**). In addition, treatment of a heart cell line led to the upregulation of four specific genes which were related to heart failure and cardiovascular inflammation and events. These four genes *CAMTA2, ITGB6, NFATc2*, and *XDH* are all related to heart failure, fibrosis, and cardiovascular disease. *CAMTA2* is in the family of transcriptional coactivators which promote cardiac hypertrophy where the AAbs might activate protein kinase D leading to activation of CAMTA2 (77); *ITGB6*, the gene for the integrin beta 6, can be activated by TGF-beta and SMAD signaling to promote fibrosis (78); NFATc2 (nuclear factor of activated T cells) is a necessary mediator of the phosphatase calcineurin-dependent cardiac hypertrophy and a major intracellular signaling pathway in pathological cardiac remodeling and heart failure (79); and XDH, the gene for xanthine oxidoreductase (XOR), promotes chronic inflammation and oxidative stress associated with vascular damage (80). In a prospective study that included 257 patients with heart failure with preserved ejection fraction and a median follow-up period of 809 days, high XOR activity was suggested to be an independent risk factor for major adverse cardiovascular events (81).

The potential role of CM AAbs in poor outcomes is supported in several other studies, including one of a myocarditis cohort (82), and another in cardiac events in type 1 diabetes (44). Since CM AAbs are not currently used as a biomarker for poor outcomes, studies of larger cohorts are needed to further support this hypothesis. Our findings support data presented by Simpson and Cantor, et al. who found significantly higher CM AAbs in baseline serum samples from newly diagnosed groups of pediatric myocarditis/DCM patients compared to healthy subjects, where a higher PKA activation at the early time point was found in pediatric patients who did not recover later in disease (83). The hypothesis that the βAR contributes to poor outcomes has been supported in animal studies (18) and was recently tested in a model of PD-1 related myocarditis using repeated adrenergic stress- induced myocarditis in PD-1 deficient mice (84).

The clinical significance of AAbs to the β1AR have been recognized with the finding that the second extracellular loop has B and T cell epitopes (85) and that AAbs to this domain have potential agonistic activity in myocarditis and other cardiovascular diseases (86). Several other studies of non-specific immunoadsorption demonstrated that IgG removal improved cardiac function which coincided with reduction in β1AR AAb titers (87, 88) and cardiac improvements persisted to one year after immunoadsorption treatment (89). Patients with β1AR AAbs have benefited from both non-specific and β1-specific immunoadsorption (90); however, multiple studies have shown that β1AR AAbs reappear in some patients, coinciding with worsening of cardiac function (90, 91). Similarly, passive serum transfer studies demonstrated that β1AR AAbs have the potential to induce cardiomyopathy in the absence of other infectious or immune challenge (18). Physiologic β1AR signaling in the heart via catecholamines promotes contractility and heart rate, but overstimulation induces apoptosis (14, 92) and heart failure in murine models (93, 94).

Studies of the nervous and immune systems have shown that lymphocytes can also express the beta receptors which may also be controlled by catecholamines (95, 96). Recently, βAR signaling and T lymphocyte-produced catecholamines have been shown to be necessary for IL17A synthesis by Case et al. (96). These more recent studies connect the Th17 response potentially with PKA activation and signaling where the catecholamine norepinephrine may be regulating Th17 responses. It is possible that the CM AAbs may signal the beta receptors on Th17 cells regulating them and inducing catecholamine production. This is a question for future studies of pathogenic mechanisms in heart disease.

**Figure 6:**
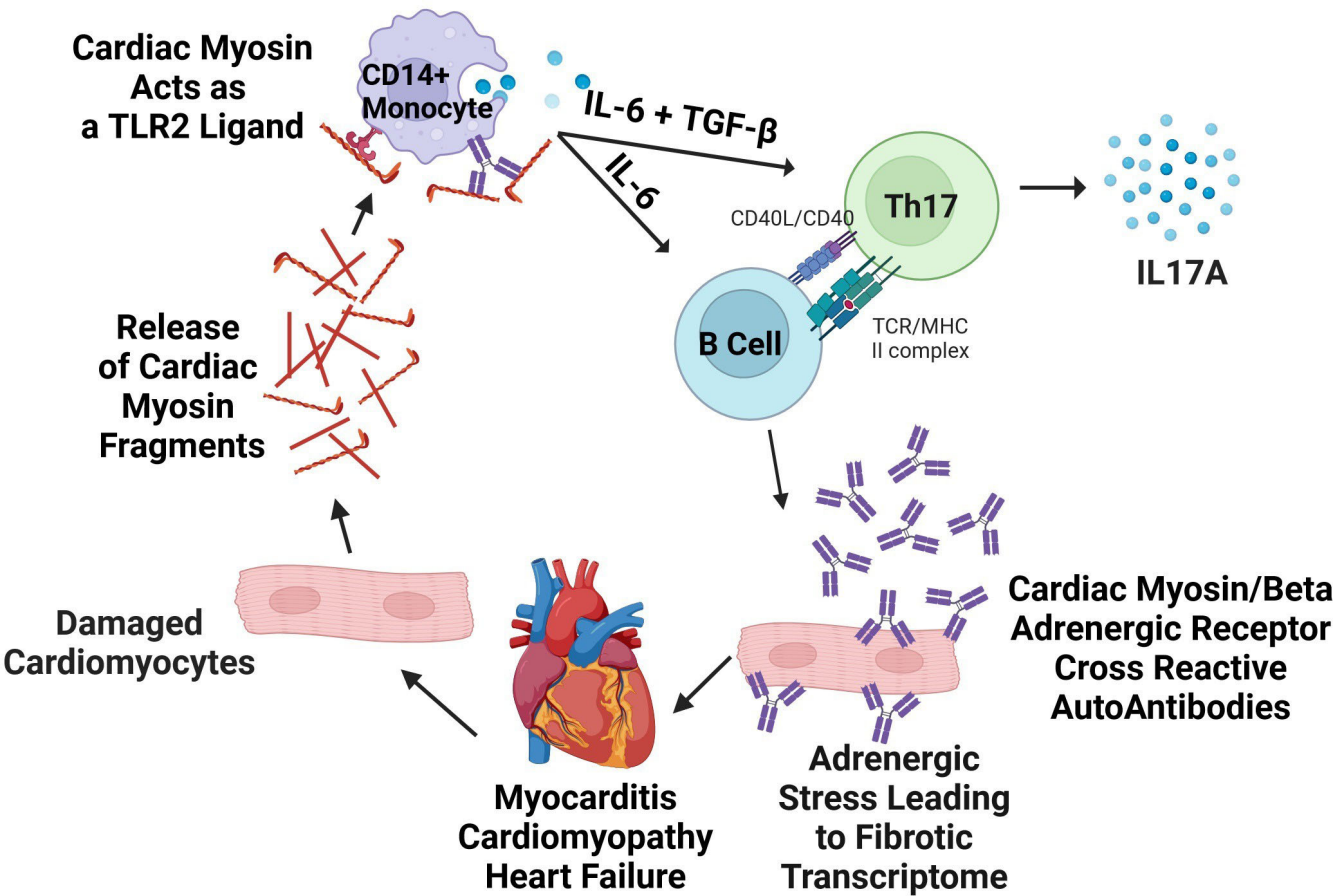
Model of pathogenic mechanisms in human autoimmune myocarditis. Lytic viral infection of cardiomyocytes or other causes of cell damage releases cardiac myosin (CM) which acts as a danger signal via TLR2/8, causing cytokine secretion that can be suppressed with anti-human TLR2 blocking antibody *in vitro*. In human myocarditis, cytokine secretion responses from CD14+ monocytes are exaggerated in myocarditis in comparison to healthy controls and promote Th17 differentiation. Antibodies to CM are also produced, and agonize βARs on cardiomyocytes or cardiac fibroblasts directly inducing a pro-fibrotic response in the myocardium. Created with BioRender.com and modified from Myers 2016 (6).

Clearly, from our previous studies, Th17 responses promote heart failure in human myocarditis with IL17A presenting as one of the two elevated biomarker cytokines in non-recovery of normal ejection fraction (6). In our previous longitudinal study, elevated IL17A production associated with non-recovery of normal EF in myocarditis patients who did not recover normal EF. The elevated cytokine biomarkers IL17A and IL-6 associated with non-recovery in the previous study (6), and the IL-6 is known to associate with and promote antibody responses which is what was found in our current report. The model of the production of AAbs emanating from the Th17 pathway is shown in **Figure 6**. The model shows cytokines IL-6 and TGF-beta stimulate Th17 cells in the Th17 pathway where IL-6 also drives B cells to make antibodies. The Th17 pathway is the major pathway for controlling extracellular pathogens such as in many upper respiratory infections which affect humans. Th17 CD4+ T cells help B cells produce antibodies which protect against pathogens, while in susceptible hosts unwanted immune responses attack the heart in autoimmune disease states (97, 98). The Th17 pathway is programmed through these cytokines to lead to antibodie*s* which deposit on microbes or in tissues and neutrophils migrate to these sites of antibody deposition. Th17 cells are clearly present in heart tissues in both autoimmune myocarditis and streptococcal induced rheumatic carditis (6, 51, 99).

Previous work in our laboratory in the Lewis rat model of EAM supported the Th17 driven pathway in myocarditis, and demonstrated AAbs developed against the CM and βAR molecules which concomitantly induced PKA signaling in cardiomyocytes (14). Other studies, including work from Caforio et al. (100) and de Leeuw et al. (101) suggested that CM AAbs are potentially early markers of idiopathic cardiomyopathy. Our previous work on CM AAbs from human myocarditis led us to hypothesize that they may contribute to poor outcomes in non-recovery and may be a useful prognostic biomarker.

In our current cohort, myocarditis patients had significantly elevated CM AAbs relative to healthy subjects at baseline (**Figure 1A**) and AAbs to CM negatively correlated with EF in non-recovered patients with poor EF outcomes at 1 year (**Figure 1D**). Importantly, AAbs to CM did not correlate with EF in patients who recovered (**Supplementary Figure 2**). We then tested sera from our cohort using 25-mer synthesized peptides from the most immunogenic region (S2 fragment) of the human CM molecule (20, 102). Using this approach, myocarditis patients who failed to recover EF by one year had CM AAbs significantly elevated at baseline compared with patients who recovered by one year (**Figure 1C**). Furthermore, CM AAbs in myocarditis sera reacted with several CM S2 peptides, synthesized from the S2 hinge fragment region of the CM molecule (**Supplementary Table 1C**). The myocarditis sera overall reacted significantly more with the CM S2 peptides compared to healthy subjects (**Figure 2**). Importantly, non-recovered patients recognized unique CM peptides 1, 3, 17, 25, and 29, which were significantly elevated in our present cohort. We observed notable similarity in peptide reactivity in a previous cross-sectional study using the same 25-mer S2 peptides examining myocarditis, DCM, and diabetic cardiomyopathy patients (20). In the work by Mascaro-Blanco et al., autoantibody epitopes for CM were identified as S2 peptides 1, 9, 17, and 30 in myocarditis or DCM, and S2 peptides 6, 8, 10 and 28 in diabetic cardiomyopathy.

The antibody cross-reactivity between the beta receptor and CM suggests that they have shared amino acid sequence homologies. **Supplementary Table 1A** shows the peptide amino acid sequences from the CM S2 fragment hinge region of human CM and their locations in myosin 7, the CM complete molecular sequence. **Supplementary Table 1B** provides the complete amino acid sequence of human cardiac myosin with the sequences recognized by the non-recovered myocarditis sera underlined and highlighted in gray. Sequence alignment between the S2 25-mer peptides used in our ELISA of myocarditis serum antibody reactivity and β1AR and B2AR revealed that an amino acid sequence of CM corresponding to the myocarditis serum highly reactive S2-3 peptide (MYH7 amino acid sequence VQAEQDNL) had a 62.5% overlap with a sequence within the third extracellular loop of βAR2 (**Supplementary Tables 1B**, **4**). Similarly, peptide S2-30 (MYH7 amino acid sequence RSKAEETQRSVND) shared 6/13 (46%) homology with the sequence RAESDEARRCYND occurring in the β1AR second extracellular (EC) loop (**Supplementary Tables 1B**, **4**). This amino acid sequence of the β1AR produced an autoimmune response specific to only the β1AR and created a positive chronotropic effect when immunized in Wistar rats (103). Additionally, AAbs to the second EC loop of the β1AR predicted death of patients with idiopathic DCM (104).

While both recovered and non-recovered patients had elevated PKA activity, it was not statistically elevated in non-recovery compared to recovery (**Figure 3B**), contrasting with significantly elevated AAbs to CM peptides in non-recovery (**Figure 1C**) and correlated AAbs to CM peptides with EF in these patients (**Figure 1D**). AAbs specific for CM have been established to cross-react with βARs and induce PKA signaling (14), however AAb interactions with GPCRs goes beyond direct agonization, and includes allosteric inhibition or activation of the endogenous ligand, alteration of Gα subunits, and other mechanisms outside the scope of this discussion but reviewed recently (105). Further, AAbs to β1AR have also been shown to sensitize the β1AR to agonization by isoproterenol by a factor of 3 (106), therefore, poor outcomes may be a result of modulation of the β1AR signaling by the AAbs which function similar to isoproterenol. A similar effect has been observed for the dopamine receptor D1R whereby AAbs against D1R sensitize it to the activation by dopamine (107).

Another contribution of CM AAbs to poor outcomes is their potential cross-reactivity with other targets. We used basic local alignment search tool (BLAST) to screen for similar sequences to the S2 CM peptides used in our assay to identify potential cross-reactive targets. Homologous amino acid sequences were considered potential targets if they were part of an extracellular domain, had 5 or more homologous amino acid residues, were expressed in relevant heart cell types (https://www.proteinatlas.org) (108), and lastly if the homologous amino acid sequence was a predicted B cell epitope (109). These predicted B cell epitopes are presented in **Supplementary Table 5**.

Our analysis of transcriptomic changes in H9c2 cells after stimulation with myocarditis patient-derived human mAb 2C.4 showed a marked increase in fibrosis-associated pathways (**Figures 4**, **5**). We chose the H9c2 primary heart cell line anticipating an effect of mAb 2C.4 due to autoantibody overstimulation of βARs which is known to lead to apoptosis (14, 20). H9c2 cells have in the past been a useful model for fibrosis given they can secrete collagens demonstrated by a proteomic analysis of H9C2 which found Col1a1, Col1a2, and Col3a1 (110), which are ordinarily found in fibroblasts (111). β2AR signaling on fibroblasts was shown to induce proliferation and production of IL-6 (112), and our analysis of H9c2 cells demonstrates an upregulation of fibrosis transcripts, therefore it is reasonable to speculate that because CM AAbs have affinity to βAR (**Supplementary Figure 1**), they may exacerbate progressive fibrosis by inducing extracellular matrix secretion, proliferation of fibroblasts, or stimulation of collagen production in myofibroblasts. The transcriptomic signature from both the human myocarditis derived mAb and the myocarditis derived sera is supported by this description of fibrosis proteins/genes. Pathogenic CM AAbs may correlate with higher CM AAb avidity (113), potentially leading to fibrosis and extracellular matrix secretion in myoblast or fibroblast cell types expressing βARs (20, 52). The transcriptomic changes induced in H9c2 cells by mAb 2C.4 within one hour of treatment mimicked that of ISO induced transcripts in cellular respiration and fibrosis-related pathways (**Figure 5**). β1AR signaling is well known to increase cellular respiration in multiple cell types (114) and has been previously demonstrated to contribute to fibrosis (115). To our knowledge, our report is the first direct demonstration of a myocarditis/DCM patient-derived human mAb driving a profibrotic signature.

When we compared our gene expression results from patient sera with that of H9c2 cardiomyocytes we found that most DE genes (238 of 259) induced by myocarditis/DCM patient sera were also DE by mAb 2C.4 and had the same direction of change (**Figure 4A**). The fact that AAbs in patient sera elicited a similar effect as human myocarditis-derived mAb 2C.4 suggests our results may have relevance to human disease. We hypothesize that CM AAbs contribute to pathogenesis through their cross-reactivity to βARs, where βAR signaling is pleiotropic, including cytotoxic and fibrotic events physiologically altering cardiomyocytes (14, 116), or causing arrhythmias (117, 118), thereby contributing to poor outcomes. Previous studies have suggested that anti-β1AR AAbs may lead to poor outcomes including myocardial scarring, cardiomyocyte toxicity, and heart failure due to excessive stimulation of the βAR (18, 119). CM AAbs may deposit in the heart or circulate and activate macrophages and monocytes through TLRs (120) and/or through FcRs. Other lines of evidence support this hypothesis such as mice lacking a functional TLR2 gene had diminished cardiac fibrosis after myocardial infarction (121, 122). Further, and most importantly, we have shown that CM is a TLR2 ligand (120).

There are several limitations to our study. It is known that many cell types may express beta receptors at different levels (123, 124) and in our study we wanted to consider the cardiomyocyte and the cardiac fibroblast as targets of the AAbs in myocarditis/DCM. The cell line we used has characteristics of both cell types since it came from what was described as a primary heart cell. The cell line has features of myoblast with cardiac like phenotype (125, 126) but also extracellular matrix secretion properties of a fibroblast. The H9c2 cell line, while primarily considered a cardiac muscle cell line derived from embryonic rat heart, can exhibit some characteristics similar to fibroblasts under specific conditions, particularly when studying aspects like extracellular matrix production or response to certain stimuli, making it a useful model for investigating cardiac fibroblast behavior in certain research contexts These features made it a suitable model for testing autoantibody effects *in vitro*, however future studies should include human Induced Pluripotent Stem Cells (hIPSC)-induced cardiomyocytes and human engineered heart tissues to further improve our understanding of CM/βAR AAbs in myocarditis and heart failure. Our small number of myocarditis sera limits the study, and further investigation of larger cohorts will be important to broadly assess the prognostic value of CM/βAR AAbs for poor outcomes, and if the mechanism of CM/βAR autoantibody pathogenesis is direct induction of fibrosis in the heart via stimulation of fibroblasts as suggested in our present report, or other mechanisms.

In a recent study of systemic lupus erythematosus purified IgG from lupus carditis was tested in a similar format to ours, where purified serum IgG from SLE patients with low abnormal <50% EF, was tested on human engineered heart tissue composed of human IPSCs cardiomyocytes and fibroblasts (127). The results found IgG AAbs bound to several heart cell surface proteins with one protein that appeared related to fibrosis in the human engineered heart tissue (127). In addition, the SLE IgG AAbs directly affected the engineered heart tissues by altering cellular composition, respiration, and calcium handling. It was interesting that they did not bind to either CM or βAR or other known targets of heart AAbs in humans (127). Future studies of human cardiomyocyte cell lines and human engineered heart tissues using IPSCs will improve our understanding of CM/βAR AAbs in myocarditis and heart failure and test the hypothesis that they may lead to fibrosis in IPSCs or engineered heart tissues containing both fibroblasts and cardiomyocyte IPSCs representing truer models of human disease (128).

Beta adrenergic (β-AR) receptors are G-protein coupled receptors which are stimulated by catecholamines epinephrine and norepinephrine (123, 124). β-AR receptors signal through cyclic adenosine monophosphate (cAMP) and cAMP-dependent PKA (123, 124). Further, β-blockers, including propranolol as used in our study, block β_1_ and β_2_ receptors, inhibiting the excitatory effect of catecholamines such as epinephrine or norepinephrine on the cardiac β_1_ receptor and that was evident when the PKA signaling by IgG antibody was blocked by propranolol. Beta blockers lower blood pressure, reduce heart rate, improve cardiac function, reduce mortality and improve quality of life in patients with HF (123, 124). Thus, identification of the presence of AAbs that may signal the beta receptor is critical to understanding the pathogenesis and treatment of myocarditis/DCM.

Since fibroblasts are generally the cell type leading to fibrosis, it is important to understand that the resident cell population in the heart maintains normal homeostasis but can become important in the induction of fibrosis in disease states. The role of βAR is not well known on fibroblasts, but studies suggest it may play a role in activation of fibroblasts to secrete cytokines such as IL-6, which is one of the 2 cytokine biomarkers we have identified as a biomarker of non-recovery in myocarditis patient blood (6). Studies have identified an important role for β2AR in regulating fibroblast proliferation through IL-6 secretion and activation of fibrosis pathways as we have found in our study. The βAR signaling component in serum of myocarditis patients, however, was not IL-6 in this study but was IgG which could be blocked in its signaling activity by anti-IgG beads. Inflammation in the heart after injury such as in a viral infection or other injury of the heart is largely mediated by pro-inflammatory cytokine release such as IL-6 by immune cells and fibroblasts, which further induce myofibroblast differentiation and fibroblast proliferation (112). β2AR activation with isoproterenol has been shown to increase dermal fibroblast migration and proliferation while inducing IL-6 production. Studies have shown that isoproterenol will activate primary murine adult and neonatal rat cardiac fibroblasts through activation of β2AR which leads to activation as well as IL-6 production.

It is possible that the activation of the βAR by AAbs leads to the IL-6 induction as well as other disease related effects that come from the Th17 pathogenesis and pathways. Our study connects the two biomarkers elevated in non-recovery with the AAbs that react with both CM and the βAR (**Figure 6**). Importantly, βAR are important regulators of cardiac function as well as pathological conditions and represent a major therapeutic target for myocarditis, DCM, and heart failure. The β1AR is expressed in the heart on cardiomyocytes where it has been extensively studied for its role in regulating contractility, cell growth, and survival (123, 124). The β2AR is prevalent on other cell populations in the heart including immune cells, endothelial cells, and fibroblasts (112, 123, 124). Our small number of myocarditis sera limits the study, and further investigation of larger cohorts will be important to broadly assess the prognostic value of CM/βAR AAbs for poor outcomes, and if the mechanism of CM/βAR autoantibody pathogenesis is direct induction of fibrosis in the heart via stimulation of fibroblasts as suggested in our present report, or other mechanisms.

There are few longitudinal myocarditis studies that have investigated biomarkers to identify recovered and non-recovered groups, and ours has provided evidence supporting the potential pathogenic role of anti-cardiac autoantibodies. Studies by Caforio et al. and Schultheiss et al. are the most notable that support our findings (11, 129), however no other studies have connected the pathogenic relationship of CM autoantibodies with βAR agonization leading to fibrosis as we have suggested here. Our model of anti-cardiac autoimmunity proposes Th17 pathogenesis is connected with AAbs to CM signaling βARs due to molecular mimicry and is vital for complete understanding of pathogenesis in autoimmune heart diseases including myocarditis, and imperative to understand how inflammation and fibrosis can be therapeutically controlled early in the disease state.

## Data Availability

Sequencing data can be accessed through NIH National Center for Biotechnology Information under BioProject accession PRJNA1025010.
