# Supplementary material for: Cardiac autoantibodies promote a fibrotic transcriptome and reduced ventricular recovery in human myocarditis
Source: Front Immunol. 2025 Mar 20;16:1500909. doi: 10.3389/fimmu.2025.1500909 (PMC11965655; doi:10.3389/fimmu.2025.1500909)
Supplement: Supplementary file 1 [file DataSheet1.pdf]

Supplementary Figure 1: Myocarditis/DCM-derived monoclonal antibodies selected for strong reactivity to B1AR, B2AR, and CM. After several rounds of selection and screening, we arrived at several B-cell hybridoma clones (1C.3, 2C.1, 2C.3, and 2C.4) with strong reactivity to both β1 and β2 adrenergic receptors and CM. It did not react with skeletal myosin, collagen, or BSA. Our present study used human mAb 2C.4 for its greatest reactivity in the ELISA to human cardiac myosin and the two βARs 1 and 2.

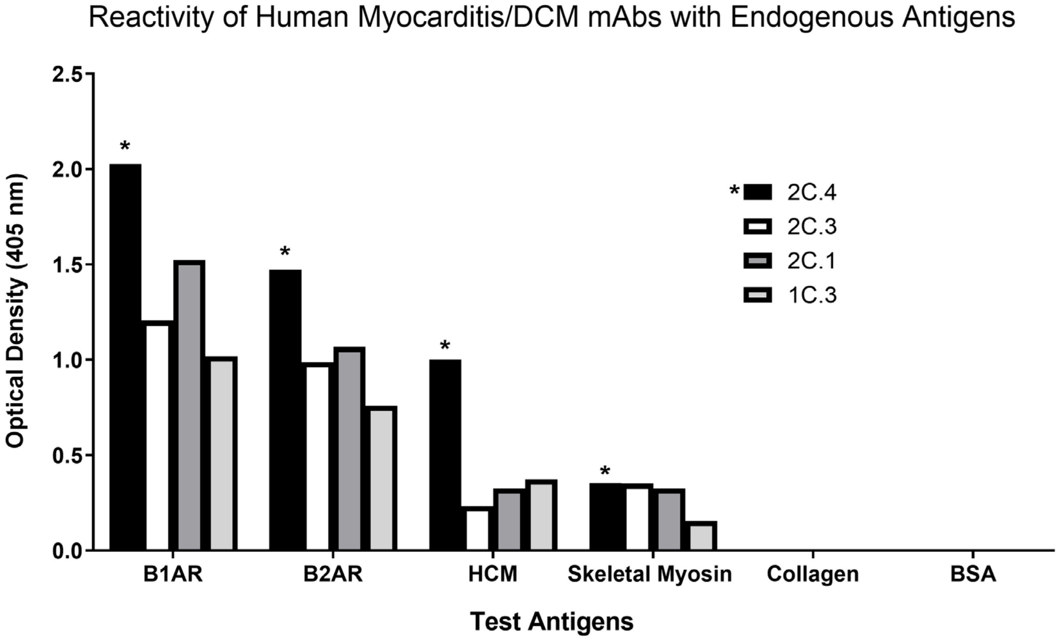

Supplementary Figure 2: LVEF does not correlate with CM autoantibody titers in recovered myocarditis patients.

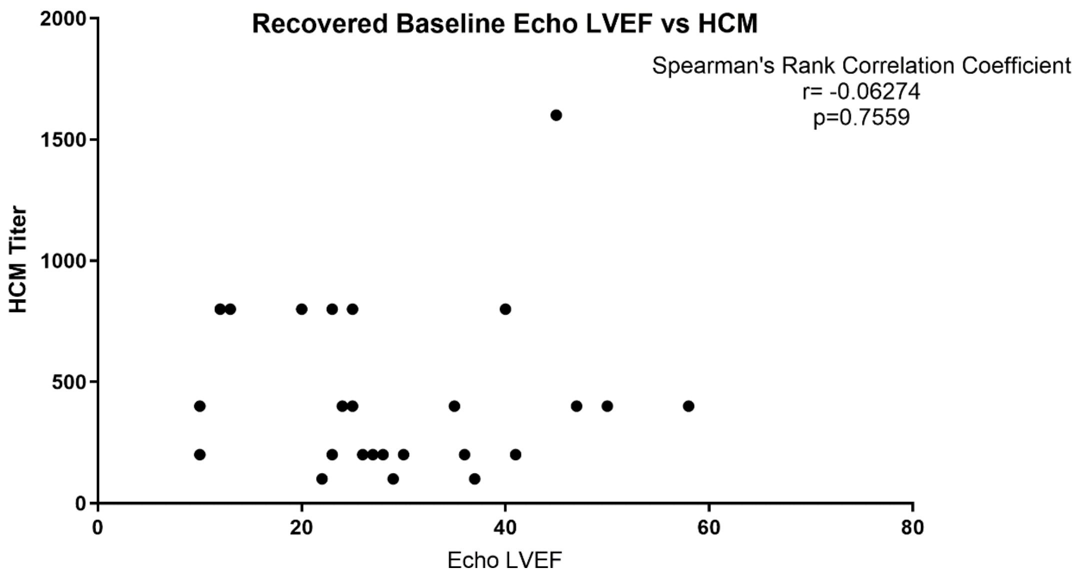

Supplementary Figure 3: LVEF Correlates with CM AAb titers in non-recovered (n=5) patients (one outlier removed).

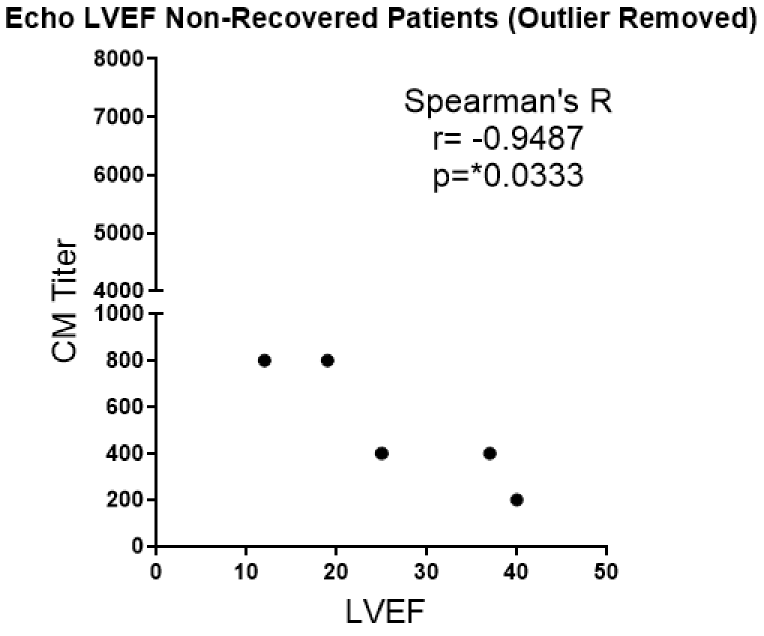

Supplementary Figure 4: PKA activation by myocarditis sera in an antibody-dependent βAR-dependent manner. Our previously published work demonstrated that CM autoantibodies cross-react with βARs and activate PKA (1-3). Here we performed an inhibition analysis (n=3) to demonstrate that myocarditis sera IgG induces PKA signaling and that PKA activation is inhibited by absorption of the sera with anti-IgG beads but not anti-BSA beads. PKA signaling is diminished by pharmacological inhibition of βARs by the beta blocker propranolol.

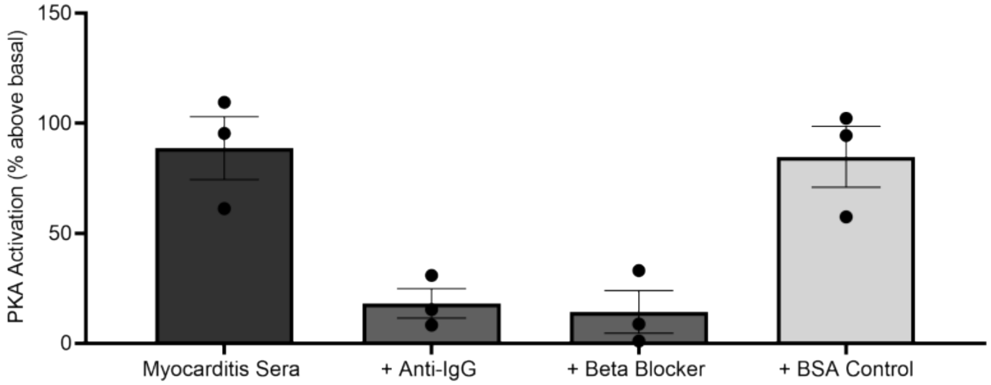

Supplementary Figure 5 (BELOW): Analysis of DE genes with STRINGdb Shows Increased Fibrosis Pathways in H9C2 Cells After Treatment with mAb 2C.4 and Isoproterenol. H9C2 cells were treated with either myocarditis/DCM-derived human mAb 2C.4 or isoproterenol, and differential gene expression (DE) analysis was performed with DESeq2. DE genes were used in overrepresentation analysis in STRINGdb (https//string-db.org) in a second, parallel analysis to our GSEA analysis. STRINGdb analysis confirmed the presence of a cluster of fibrosis-associated pathways in both treatment conditions (**Supplementary Figures 5A** and **5C**). Likewise, a cluster of mitochondrial respiration pathways were observed in both treatment conditions (**Supplementary Figures 5B** and **5D**). **Supplementary Tables 2A-2D** give the description of the pathway, database, strength and FDR for the pathway enrichment, as well as the proteins in our analysis that drove the association and contain the results from statistical analysis. **Supplementary Tables 3A** and **3B** give the input gene list used in this analysis, that was obtained from differential expression analysis with DESeq2.

Supplementary Figure 5A: mAB vs Basal: STRINGdb Fibrosis Cluster

(Pathways shown in Supplementary Table 1A. Comparing Supplementary Tables 2A and 2C shows similar pathways)

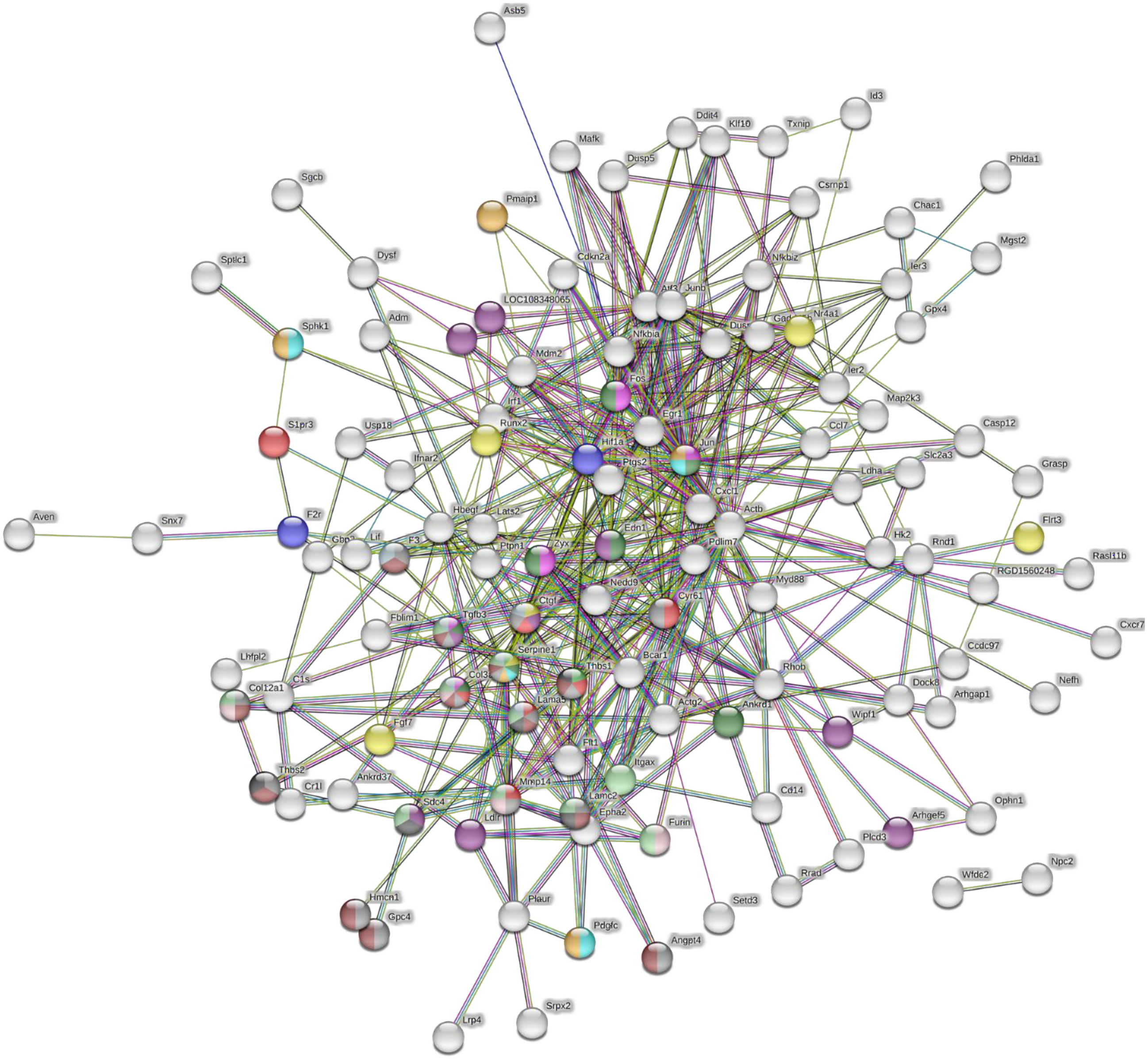

Supplementary Figure 5B: mAB vs Basal: STRINGdb Respiratory Cluster (Pathways shown in Supplementary Table 1B. Comparing Supplementary Tables 2B and 2D shows similar pathways)

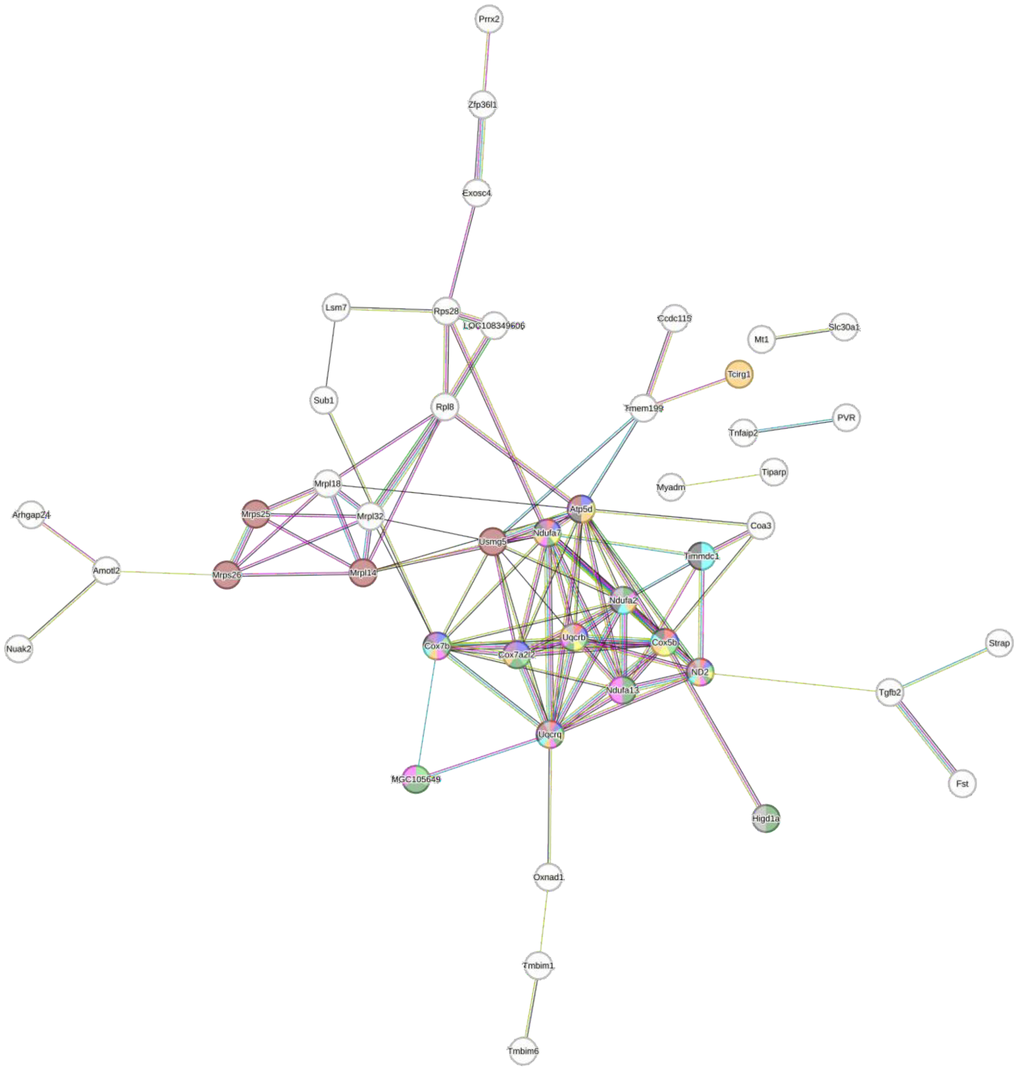

Supplementary Figure 5C: Isoproterenol vs Basal: STRINGdb Fibrosis Cluster (Pathways shown in Supplementary Table 1C. Comparing Supplementary Tables 2A and 2C shows similar pathways)

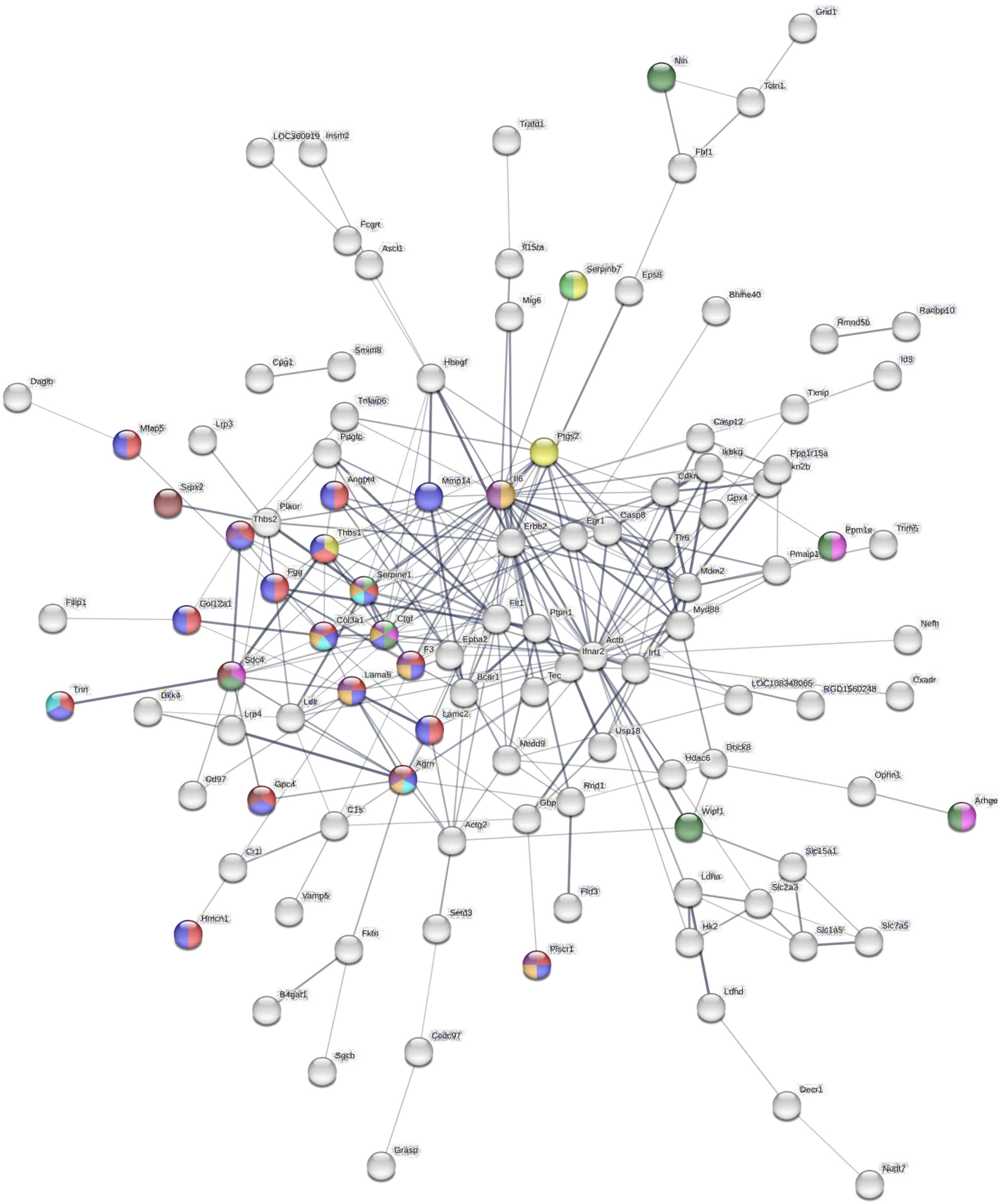

Supplementary Figure 5D: Isoproterenol vs Basal: STRINGdb Respiratory Cluster (Pathways shown in Supplementary Table 1D. Comparing Supplementary Tables 2B and 2D shows similar pathways)

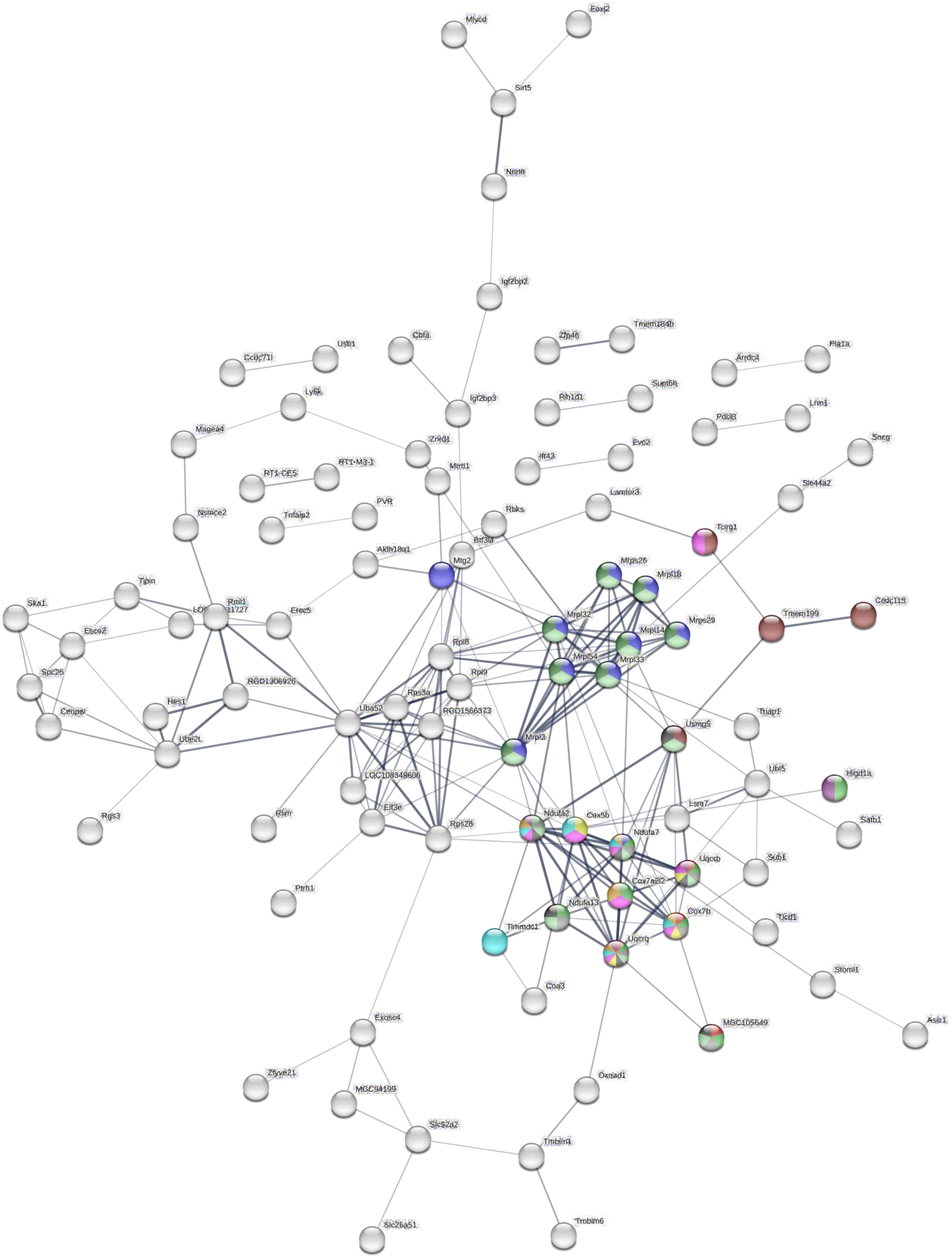

Supplementary Table 1A: Human Cardiac S2 Peptide Sequences and Location in MYH7 #

Peptide	Amino Acid Sequence	Location in MYH7
S2-1	SAEREKEMASMKEEFTRLKEALEKS	(aa 842-866)*^
S2-2	FTRLKEALEKSEARRKELEEKMVSL	(aa 856-880)
S2-3	RKELEEKMVSLLQEKNDLQLQVQAE	(aa 870-894)^
S2-4	KNDLQLQVQAEQDNLADAEERCDQL	(aa 881-908)
S2-5	LADAEERCDQLIKNKIQLEAKVKEM	(aa 898-922)
S2-6	KIQLEAKVKEMNERLEDEEEMNAEL	(aa 912-936)
S2-7	LEDEEEMNAELTAKKRKLEDECSEL	(aa 926-950)
S2-8	KRKLEDECSELKRDIDDLELTLAKV	(aa 940-964)
S2-9	IDDLELTLAKVEKEKHATENKVKNL	(aa 954-978)
S2-10	KHATENKVKNLTEEMAGLDEIIAKL	(aa 968-992)
S2-11	MAGLDEIIAKLTKEKKALQEAHQQA	(aa 982-1006)
S2-12	KKALQEAHQQALDDLQAEEDKVNTL	(aa 997-1020)
S2-13	LQAEEDKVNTLTKAKVKLEQQVDDL	(aa 1010-1034
S2-14	KVKLEQQVDDLEGSLEQEKKVRMDL	(aa 1024-1048)
S2-15	LEQEKKVRMDLERAKRKLEGDLKLT	(aa 1038-1062)
S2-16	KRKLEGDLKLTQESIMDLENDKQQL	(aa 1052-1076)
S2-17	IMDLENDKQQLDERLKKKDFELNAL	(aa 1066-1090)^
S2-18	LKKKDFELNALNARIEDEQALGSQL	(aa 1080-1104)
S2-19	IEDEQALGSQLQKKLKELQARIEEL	(aa 1094-1118)
S2-20	LKELQARIEELEEELESERTARAKV	(aa 1108-1132)
S2-21	LESERTARAKVEKLRSDLSRELEEI	(aa 1122-1146)
S2-22	RSDLSRELEEISERLEEAGGATSVQ	(aa 1136-1160)
S2-23	LEEAGGATSVQIEMNKKREAEFQKM	(aa 1150-1174)
S2-24	NKKREAEFQKMRRDLEEATLQHEAT	(aa 1164-1188)
S2-25	LEEATLQHEATAAALRKKHADSVAE	(aa 1178-1202)^
S2-26	LRKKHADSVAELGEQIDNLQRVKQK	(aa 1192-1216)
S2-27	QIDNLQRVKQKLEKEKSEFKLELDD	(aa 1206-1230)
S2-28	EKSEFKLELDDVTSNMEQIIKAKAN	(aa 1220-1244)
S2-29	NMEQIIKAKANLEKMCRTLEDQMNE	(aa 1234-1258)^
S2-30	MCRTLEDQMNEHRSKAEETQRSVND	(aa 1248-1272)
S2-31	KAEETQRSVNDLTSQRAKLQTENGE	(aa 1262-1286)
S2-32	ETQRSVNDLTSQRAKLQTENGELSR	(aa 1265-1289)

link to sequence: https://www.uniprot.org/uniprotkb/P12883/entry#sequences
*aa = amino acid residues in human cardiac myosin MYH7
^ = Significant peptides in non-recovered patient sera

Supplementary Table 1B: MYH7 Complete Amino Acid Sequence

MGDSEMAVFGAAAPYLRKSEKERLEAQTRPFDLKKDVFVPDDKQEFVKAKIVSREGGKVTAETEYGKTVTVK
EDQVMQQNPPKFDKIEDMAMLTFLHEPAVLYNLKDRYGSWMIYTYSGLFCVTVNPYKWLPVYTPEVVAAYRG
KKRSEAPPHIFSISDNAYQYMLTDRENQSILITGESGAGKTVNTKRVIQYFAVIAAIGDRSKKDQSPGKGTL
EDQIIQANPALEAFGNAKTVRNDNSSRFGKFIRIHFGATGKLASADIETYLLEKSRVIFQLKAERDYHIFYQ
ILSNKKPELLDMLLITNNPYDYAFISQGETTVASIDDAEELMATDNAFDVLGFTSEEKNSMYKLTGAIMHFG
NMKFKLKQREEQAEPDGTEEADKSAYLMGLNSADLLKGLCHPRVKVGNEYVTKGQNVQQVIYATGALAKAVY
ERMFNWMVTRINATLETKQPRQYFIGVLDIAGFEIFDFNSFEQLCINFTNEKLQQFFNHHMFVLEQEEYKKE
GIEWTFIDFGMDLQACIDLIEKPMGIMSILEEECMFPKATDMTFKAKLFDNHLGKSANFQKPRNIKGKPEAH
FSLIHYAGIVDYNIIGWLQKNKDPLNETVVGLYQKSSLKLLSTLFANYAGADAPIEKGKGKAKKGSSFQTVS
ALHRENLNKLMTNLRSTHPHFVRCIIPNETKSPGVMDNPLVMHQLRCNGVLEGIRICRKGFPNRILYGDFRQ
RYRILNPAAIPEGQFIDSRKGAEKLLSSLDIDHNQYKFGHTKVFFKAGLLGLLEEMRDERLSRIITRIQAQS
RGVLARMEYKKLLERRDSLLVIQWNIRAFMGVKNWPWMKLYFKIKPLLKSAEREKEMASMKEEFTRLKEALE
KSEARRKELEEKMVSLLQEKNDLQLQVQAEQDNLADAEERCDQLIKNKIQLEAKVKEMNERLEDEEEMNAEL
TAKKRKLEDECSELKRDIDDLELTLAKVEKEKHATENKVKNLTEEMAGLDEIIAKLTKEKKALQEAHQQALD
DLQAEEDKVNTLTKAKVKLEQQVDDLEGSLEQEKKVRMDLERAKRKLEGDLKLTQESIMDLENDKQQLDERL
KKKDFELNALNARIEDEQALGSQLQKKLKELQARIEELEEELEAERTARAKVEKLRSDLSRELEEISERLEE
AGGATSVQIEMNKKREAEFQKMRRDLEEATLQHEATAAALRKKHADSVAELGEQIDNLQRVKQKLEKEKSEF
KLELDDVTSNMEQIIKAKANLEKMCRTLEDQMNEHRSKAEETQRSVNDLTSQRAKLQTENGELSRQLDEKEA
LISQLTRGKLTYTQQLEDLKRQLEEEVKAKNALAHALQSARHDCDLLREQYEEETEAKAELQRVLSKANSEV
AQWRTKYETDAIQRTEELEEAKKKLAQRLQEAEEAVEAVNAKCSSLEKTKHRLQNEIEDLMVDVERSNAAAA
ALDKKQRNFDKILAEWKQKYEESQSELESSQKEARSLSTELFKLKNAYEESLEHLETFKRENKNLQEEISDL
TEQLGSSGKTIHELEKVRKQLEAEKMELQSALEEAEASLEHEEGKILRAQLEFNQIKAEIERKLAEKDEEME
QAKRNHLRVVDSLQTSLDAETRSRNEALRVKKKMEGDLNEMEIQLSHANRMAAEAQKQVKSLQSLLKDTQIQ
LDDAVRANDDLKENIAIVERRNNLLQAELEELRAVVEQTERSRKLAEQELIETSERVQLLHSQNTSLINQKK
KMDADLSQLQTEVEEAVQECRNAEEKAKKAITDAAMMAEELKKEQDTSAHLERMKKNMEQTIKDLQHRLDEA
EQIALKGGKKQLQKLEARVRELENELEAEQKRNAESVKGMRKSERRIKELTYQTEEDRKNLLRLQDLVDKLQ
LKVKAYKRQAEEAEEQANTNLSKFRKVQHELDEAEERADIAESQVNKLRAKSRDIGTKGLNEE

Supplementary Table 1C: Diagram of Cardiac Myosin (CM) Molecule (highlighting the proteolytic fragments including the S2 Hinge Region Fragment of CM from which we made the series of S2 Peptides). Molecular weight of each proteolytic fragment is shown in parentheses in the diagram (4) .

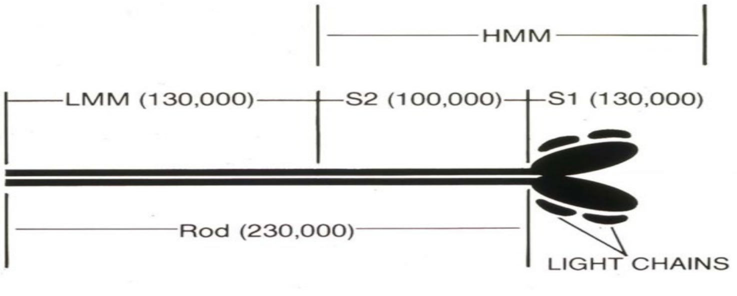

Diagram of the myosin molecule.

HMM, Heavy Meromyosin; LMM, Light Meromyosin; S2 (Hinge) and S1 (Head) proteolytic fragments

Supplementary Table 2A: mAB vs Basal: Selected Pathways in STRINGdb Fibrosis Cluster

#color	category	term ID	term description	OBS gene count	BG gene count	strength	FDR	matching proteins in your network (labels)
lightblue	COMPARTMENTS	GOCC:0062023	Collagen-containing extracellular matrix	6	131	0.89	1.43E-02	Serpine1,Col3a1,Lama5,Tgfb3,F3,Ctgf
grey	GO Component	GO:0031012	Extracellular matrix	17	445	0.81	3.35E-06	Serpine1,Gpc4,Col3a1,Angpt4,Lama5,Tgfb3,Thbs2,F3,Olfml2a,Cyr61,Ctgf,Tsku,Hmcn1,Lamc2,Col12a1,Mmp14,Thbs1
maroon	GO Component	GO:0062023	Collagen-containing extracellular matrix	12	317	0.81	2.50E-04	Serpine1,Gpc4,Col3a1,Angpt4,Lama5,Tgfb3,Thbs2,F3,Hmcn1,Lamc2,Col12a1, Thbs1
red	GO Function	GO:0005178	Integrin binding	7	168	0.85	2.02E-02	Col3a1,Lama5,S1pr3,Cyr61,Ctgf,Mmp14, Thbs1
black	KEGG	rno04512	ECM-receptor interaction	5	78	1.04	1.40E-03	Lama5,Thbs2,Sdc4,Lamc2,Thbs1
darkgreen	GO Process	GO:0071560	Cellular response to transforming growth factor beta stimulus	8	161	0.93	4.20E-04	Serpine1,Col3a1,Fos,Jun,Tgfb3,Edn1, Zyx,Ankrd1
yellow	GO Process	GO:0044344	Cellular response to fibroblast growth factor stimulus	6	88	1.06	1.00E-03	Serpine1,Flrt3,Nr4a1,Fgf7,Ctgf,Runx2
magenta	GO Process	GO:0007179	Transforming growth factor beta receptor signaling pathway	5	84	1	6.90E-03	Col3a1,Fos,Jun,Tgfb3,Zyx
blue	GO Process	GO:0002248	Connective tissue replacement involved in inflammatory response wound healing	2	5	1.83	2.12E-02	Hif1a,F2r
purple	GO Process	GO:1902903	Regulation of supramolecular fiber organization	9	410	0.57	2.42E-02	LOC108348065,Ldlr,Tgfb3,Edn1,Sdc4,Ctgf,Wipf1,Arhgef5,Hdac6
orange	GO Process	GO:0048145	Regulation of fibroblast proliferation	5	123	0.84	2.70E-02	Serpine1,Jun,Pdgfc,Pmaip1,Sphk1
limegreen	GO Process	GO:0051918	Negative regulation of fibrinolysis	2	7	1.69	3.27E-02	Serpine1,Thbs1
cyan	GO Process	GO:0048146	Positive regulation of fibroblast proliferation	4	80	0.93	3.95E-02	Serpine1,Jun,Pdgfc,Sphk1
lightgreen	Reactome	RNO-1474244	Extracellular matrix organization	10	255	0.82	1.60E-03	Serpine1,Col3a1,Lama5,Tgfb3,Furin,Sdc4,Lamc2,Col12a1,Itgax,Mmp14
pink	Reactome	RNO-1442490	Collagen degradation	4	45	1.18	4.73E-02	Col3a1,Furin,Col12a1,Mmp14

Supplementary Table 2B: mAB vs Basal: Selected Pathways in STRINGdb Respiratory Cluster

#color	category	term ID	term description	Obs. gene count	BG gene count	strength	FDR	matching proteins in your network (labels)
maroon	COMPARTMENTS	GOCC:0098798	Mitochondrial protein complex	7	141	1.15	1.60E-03	Mrps25,Atp5d,Mrpl14,Usmg5,Mrps26,ND2,Uqcrq
darkgreen	GO Component	GO:0070469	Respirasome	10	117	1.39	2.18E-08	Ndufa7,Ndufa2,Uqcrb,Ndufa13, Cox7a2l2,ND2,Cox7b,Higd1a, MGC105649,Uqcrq
magenta	GO Component	GO:0098803	Respiratory chain complex	8	98	1.37	9.40E-07	Ndufa7,Ndufa2,Uqcrb,Ndufa13, ND2,Cox7b,MGC105649,Uqcrq
orange	KEGG	rno00190	Oxidative phosphorylation	10	130	1.34	2.05E-08	Ndufa7,Atp5d,Cox5b,Tcirg1,Ndufa2,Uqcrb,Cox7a2l2,ND2, Cox7b,Uqcrq
grey	UniProt Keywords	KW-0679	Respiratory chain	5	40	1.55	8.71E-05	Ndufa2,Uqcrb,ND2,Higd1a, Uqcrq
blue	GO Process	GO:0006119	Oxidative phosphorylation	8	107	1.33	1.00E-04	Ndufa7,Atp5d,Cox5b,Uqcrb,Cox7a2l2,ND2,Cox7b,Uqcrq
limegreen	GO Process	GO:0022900	Electron transport chain	8	135	1.23	2.80E-04	Ndufa7,Cox5b,Uqcrb,Cox7a2l2, ND2,Cox7b,MGC105649,Uqcrq
red	GO Process	GO:0042775	Mitochondrial atp synthesis coupled electron transport	5	60	1.37	1.58E-02	Ndufa7,Cox5b,Uqcrb,ND2, Uqcrq
yellow	GO Process	GO:0045333	Cellular respiration	6	147	1.06	3.49E-02	Ndufa7,Atp5d,Cox5b,Uqcrb, ND2,Uqcrq
black	Reactome	RNO-163200	Respiratory electron transport, ATP synthesis by chemiosmotic coupling, and heat production by uncoupling proteins.	8	116	1.29	1.72E-05	Timmdc1,Ndufa7,Atp5d,Cox5b, Ndufa2,ND2,Cox7b,Uqcrq
cyan	Reactome	RNO-611105	Respiratory electron transport	7	91	1.34	2.83E-05	Timmdc1,Ndufa7,Cox5b,Ndufa2,ND2,Cox7b,Uqcrq
purple	WikiPathways	WP59	Electron transport chain	7	87	1.36	5.90E-06	Ndufa7,Atp5d,Ndufa2,Cox7a2l2,ND2,Cox7b,Uqcrq

Supplementary Table 2C: Isoproterenol vs Basal: Selected Pathways in STRINGdb Fibrosis Cluster

#color	category	term ID	term description	OBS. gene count	BG gene count	strength	FDR	matching proteins in your network (labels)
purple	COMPARTMENTS	GOCC:0031012	Extracellular matrix	9	171	0.94	5.8E-04	Serpine1,Col3a1,Lama5,Il6,Thbs2,F3,Ctgf,Agrn,Plscr1
orange	COMPARTMENTS	GOCC:0062023	Collagen-containing extracellular matrix	8	131	1	6.5E-04	Serpine1,Col3a1,Lama5,Il6,F3,Ctgf,Agrn,Plscr1
blue	GO Component	GO:0031012	Extracellular matrix	19	445	0.84	1.1E-07	Serpine1,Gpc4,Tnn,Col3a1,Angpt4,Lama5,Thbs2,F3,Ctgf,Mfap5,Tsku,Fgg,Hmcn1,Lamc2,Col12a1,Agrn,Plscr1,Mmp14,Thbs1
red	GO Component	GO:0062023	Collagen-containing extracellular matrix	16	317	0.92	1.9E-07	Serpine1,Gpc4,Tnn,Col3a1,Angpt4,Lama5,Thbs2,F3,Mfap5,Fgg,Hmcn1,Lamc2,Col12a1,Agrn,Plscr1,Thbs1
maroon	UniProt Keywords	KW-0654	Proteoglycan	4	35	1.27	6.7E-03	Gpc4,Srpx2,Sdc4,Agrn
limegreen	GO Process	GO:0032967	Positive regulation of collagen biosynthetic process	4	31	1.32	4.0E-03	Serpine1,Serpinb7,Ctgf,Rgcc
magenta	GO Process	GO:0051496	Positive regulation of stress fiber assembly	5	63	1.11	4.0E-03	Ppm1e,Sdc4,Ctgf,Arhgef5,Rgcc
yellow	GO Process	GO:0071636	Positive regulation of transforming growth factor beta production	3	21	1.37	2.0E-02	Serpinb7,Ptgs2,Thbs1
darkgreen	GO Process	GO:1902905	Positive regulation of supramolecular fiber organization	7	214	0.73	2.0E-02	Ppm1e,Nin,Sdc4,Ctgf,Wipf1,Arhgef5,Rgcc
cyan	Reactome	RNO-3000178	ECM proteoglycans	4	38	1.24	3.4E-02	Serpine1,Tnn,Col3a1,Agrn

Supplementary Table 2D: Isoproterenol vs Basal: Selected Pathways in STRINGdb Respiratory Cluster

#color	category	term ID	term description	OBS gene count	BG gene count	strength	FDR	matching proteins in your network (labels)
lightgreen	GO Component	GO:0098798	Mitochondrial protein complex	15	325	0.93	0.000000468	Ndufa7,Mrps25,Mrpl18, Mrpl32,Ndufa2,Mrpl2,Mrpl14,Usmg5,Mrpl54,Mrps26,Mrpl33,Uqcrb,Ndufa13,MGC105649,Uqcrq
blue	GO Component	GO:0005761	Mitochondrial ribosome	10	107	1.23	0.000000501	Ndufa7,Mrps25,Mrpl18, Mrpl32,Mrpl2,Mrpl14,Mrpl54,Mrps26,Mrpl33, Mtg2
limegreen	GO Component	GO:0070469	Respirasome	9	117	1.15	0.00000528	Ndufa7,Ndufa2,Uqcrb,Ndufa13,Cox7a2l2,Cox7b,Higd1a,MGC105649, Uqcrq
grey	GO Component	GO:0098803	Respiratory chain complex	7	98	1.12	0.00018	Ndufa7,Ndufa2,Uqcrb,Ndufa13,Cox7b, MGC105649,Uqcrq
red	GO Component	GO:0070069	Cytochrome complex	4	34	1.33	0.0033	Uqcrb,Cox7b, MGC105649,Uqcrq
black	GO Component	GO:0098800	Inner mitochondrial membrane protein complex	7	172	0.87	0.0034	Ndufa7,Ndufa2,Usmg5, Uqcrb,Ndufa13, MGC105649,Uqcrq
maroon	GO Component	GO:0016469	Proton-transporting two-sector atpase complex	4	63	1.07	0.0251	Tmem199,Ccdc115, Tcirg1,Usmg5
magenta	KEGG	rno00190	Oxidative phosphorylation	8	130	1.05	0.00014	Ndufa7,Cox5b,Tcirg1,Ndufa2,Uqcrb,Cox7a2l2, Cox7b,Uqcrq
purple	UniProt Keywords	KW-0679	Respiratory chain	4	40	1.26	0.0081	Ndufa2,Uqcrb,Higd1a, Uqcrq
yellow	STRING clusters	CL:22587	Cytochrome complex, and cytochrome c oxidase subunit vb	4	32	1.36	0.013	Cox5b,Uqcrb,Cox7b, Uqcrq
darkgreen	Reactome	RNO-5389840	Mitochondrial translation elongation	8	79	1.27	0.0000193	Mrps25,Mrpl18,Mrpl32, Mrpl2,Mrpl14,Mrpl54, Mrps26,Mrpl33
cyan	Reactome	RNO-611105	Respiratory electron transport	6	91	1.08	0.0047	Timmdc1,Ndufa7,Cox5b,Ndufa2,Cox7b,Uqcrq
orange	WikiPathways	WP59	Electron transport chain	5	87	1.02	0.0216	Ndufa7,Ndufa2, Cox7a2l2,Cox7b,Uqcrq

Supplementary Table 3A: STRINGdb Input for mAb vs Basal
(Used to generate STRINGdb plots and tables)

gene	baseMean	log2FoldChange	lfcSE	stat	pvalue	padj
AABR07007121.1	75.5766	1.2129	0.3110	3.8997	0.0001	0.0015
AABR07021759.1	4615.8076	-1.6251	0.3693	-4.4001	0.0000	0.0002
AABR07031089.1	2073.3261	-8.9048	1.0678	-8.3396	0.0000	0.0000
AABR07034639.1	553.5015	2.2077	0.5263	4.1945	0.0000	0.0005
AABR07036855.1	67.7383	1.1752	0.3369	3.4888	0.0005	0.0054
AABR07037203.1	144.9665	-2.1188	0.3001	-7.0601	0.0000	0.0000
AABR07037419.1	44.0729	-7.5455	1.6072	-4.6947	0.0000	0.0001
AABR07037489.1	36.5149	1.4698	0.5107	2.8781	0.0040	0.0265
AABR07042611.1	43.9641	1.1279	0.3915	2.8809	0.0040	0.0264
AABR07049405.1	486.4439	-1.1170	0.1563	-7.1485	0.0000	0.0000
AABR07061825.1	328.4441	-6.4564	1.4650	-4.4071	0.0000	0.0002
AABR07062154.1	85.0897	-1.2972	0.3658	-3.5461	0.0004	0.0046
AC109048.1	57.2222	1.0401	0.3457	3.0089	0.0026	0.0195
AC139642.2	620.4737	1.2445	0.3252	3.8275	0.0001	0.0019
AC141489.1	836.8527	1.0643	0.1386	7.6776	0.0000	0.0000
AC141959.1	86.9369	1.0224	0.2892	3.5353	0.0004	0.0047
Ackr3	271.0893	1.0117	0.1774	5.7025	0.0000	0.0000
Actg1	294.7604	-29.9920	4.7123	-6.3646	0.0000	0.0000
Actg2	480.5310	1.0323	0.1871	5.5174	0.0000	0.0000
Acyp1	55.0920	1.0770	0.3739	2.8802	0.0040	0.0264
Adm	58.0951	1.1400	0.3367	3.3857	0.0007	0.0073
Akr1c12	32.5490	1.2799	0.4415	2.8993	0.0037	0.0253
Amotl2	130.3705	1.4262	0.3071	4.6438	0.0000	0.0001
Angpt4	55.1176	1.2151	0.3505	3.4668	0.0005	0.0058
Ankrd1	1940.0372	1.0247	0.0966	10.6047	0.0000	0.0000
Ankrd37	35.1852	1.3329	0.4484	2.9724	0.0030	0.0214
Arhgap1	30.0598	1.3141	0.4940	2.6604	0.0078	0.0427
Arhgap24	59.2240	-1.6226	0.4263	-3.8059	0.0001	0.0021
Arhgef5	32.0314	1.4638	0.4676	3.1304	0.0017	0.0145
Arrdc3	590.8087	2.6155	0.1601	16.3330	0.0000	0.0000
Asb5	138.7669	1.0243	0.2425	4.2237	0.0000	0.0005
Atf3	36.8784	1.3721	0.3980	3.4473	0.0006	0.0061
Atp5f1d	41.7430	1.0486	0.4030	2.6017	0.0093	0.0486
Atp5md	468.2547	1.2247	0.1778	6.8882	0.0000	0.0000
Aven	43.1469	1.0339	0.3954	2.6145	0.0089	0.0473
B4gat1	129.8906	1.0046	0.2353	4.2699	0.0000	0.0004
Bag5l1	51.6729	1.4906	0.4110	3.6265	0.0003	0.0036
Bcar1	102.2341	1.6871	0.3137	5.3774	0.0000	0.0000
Bhlhe40	55.3795	2.6082	0.3925	6.6457	0.0000	0.0000
Btf3l4	116.3502	1.1408	0.2708	4.2130	0.0000	0.0005
Btg2	177.1339	2.6983	0.2398	11.2507	0.0000	0.0000
C1s	173.0811	1.0377	0.2035	5.0998	0.0000	0.0000
Cabp7	27.5807	1.4770	0.5072	2.9120	0.0036	0.0248
Casp12	72.5978	1.0357	0.3390	3.0548	0.0023	0.0176
Ccdc115	51.1331	1.0305	0.3661	2.8147	0.0049	0.0306
Ccdc126	71.4178	1.1621	0.3309	3.5124	0.0004	0.0051
Ccdc71l	47.5805	-1.1390	0.4260	-2.6737	0.0075	0.0415
Ccdc97	35.1480	1.1511	0.4330	2.6584	0.0079	0.0429

Ccl7	23.9377	4.2100	0.5959	7.0645	0.0000	0.0000
Ccn1	743.1021	2.4884	0.1881	13.2258	0.0000	0.0000
Ccn2	999.2445	1.7958	0.1721	10.4325	0.0000	0.0000
Cd14	54.1048	1.0433	0.3438	3.0346	0.0024	0.0184
Cdkn2a	95.3873	1.1281	0.2844	3.9667	0.0001	0.0012
Cdkn2b	55.0758	1.0279	0.3377	3.0440	0.0023	0.0180
Cenpo	29.5908	1.1828	0.4553	2.5979	0.0094	0.0489
Chac1	152.2729	-1.5403	0.2967	-5.1907	0.0000	0.0000
Chchd5	48.3952	1.0185	0.3716	2.7408	0.0061	0.0357
Chp1	37.8236	1.8618	0.4353	4.2768	0.0000	0.0004
Clcf1	53.3726	1.3432	0.3785	3.5490	0.0004	0.0046
Clec2g	231.7265	1.0216	0.1844	5.5392	0.0000	0.0000
Coa3	43.4308	1.0611	0.3866	2.7449	0.0061	0.0353
Col12a1	50.6316	1.2168	0.3671	3.3144	0.0009	0.0089
Col3a1	6379.7175	1.0202	0.1016	10.0376	0.0000	0.0000
Cox5b	346.0402	1.1332	0.1902	5.9590	0.0000	0.0000
Cox7a2l2	275.5358	1.0186	0.1903	5.3522	0.0000	0.0000
Cox7b	436.2847	1.0011	0.1596	6.2710	0.0000	0.0000
Cr1l	164.8916	1.1021	0.2185	5.0431	0.0000	0.0000
Creld2	94.9288	1.0721	0.2905	3.6902	0.0002	0.0030
Csrnp1	83.0179	2.2320	0.3272	6.8212	0.0000	0.0000
Cxcl1	169.0918	7.1259	0.4083	17.4520	0.0000	0.0000
D2hgdh	33.9924	1.8707	0.4681	3.9965	0.0001	0.0011
Daglb	42.8348	1.2636	0.4301	2.9380	0.0033	0.0233
Dap	52.4312	1.2470	0.3974	3.1375	0.0017	0.0142
Ddit4	425.6105	1.2956	0.1491	8.6868	0.0000	0.0000
Decr1	71.4964	1.0090	0.3207	3.1467	0.0017	0.0138
Depp1	26.0559	3.3192	0.5427	6.1162	0.0000	0.0000
Dhrs7b	57.2071	1.2251	0.3480	3.5203	0.0004	0.0050
Dnajc24	24.1183	1.4420	0.5363	2.6891	0.0072	0.0401
Dock8	39.1149	1.4813	0.4328	3.4227	0.0006	0.0065
Dpcd	51.7870	1.6905	0.3882	4.3547	0.0000	0.0003
Dram1	101.3773	1.0302	0.2829	3.6419	0.0003	0.0034
Dusp1	257.9385	2.1374	0.1748	12.2258	0.0000	0.0000
Dusp5	110.4078	1.3299	0.2962	4.4894	0.0000	0.0002
Dysf	72.9762	1.0522	0.3230	3.2572	0.0011	0.0105
Edn1	26.6204	2.5664	0.5645	4.5462	0.0000	0.0001
Egln3	127.0309	1.6488	0.2537	6.4980	0.0000	0.0000
Egr1	160.3659	4.1177	0.3766	10.9338	0.0000	0.0000
Epha2	117.8874	2.1429	0.2730	7.8505	0.0000	0.0000
Eps8	29.5403	1.6022	0.5160	3.1049	0.0019	0.0154
Ermard	28.1303	1.5038	0.4752	3.1642	0.0016	0.0132
Errfi1	268.0153	1.5682	0.1939	8.0896	0.0000	0.0000
Esco2	39.8813	1.2978	0.4290	3.0255	0.0025	0.0187
Evc	43.6594	1.3010	0.4075	3.1931	0.0014	0.0123
Evc2	54.1346	1.0892	0.3528	3.0876	0.0020	0.0161
Exosc4	76.2290	1.2312	0.3034	4.0587	0.0000	0.0009
F2r	206.8732	1.1422	0.2187	5.2232	0.0000	0.0000
F3	194.1116	2.4342	0.2461	9.8919	0.0000	0.0000
Fam13b	272.0002	-1.1789	0.1929	-6.1122	0.0000	0.0000
Fblim1	28.5597	1.5340	0.5072	3.0244	0.0025	0.0188
Fcgrt	429.2497	1.1190	0.1577	7.0962	0.0000	0.0000

Fgf7	74.2637	1.1036	0.3082	3.5813	0.0003	0.0041
Flrt3	76.6740	-1.3006	0.3203	-4.0607	0.0000	0.0009
Flt1	33.2039	1.2292	0.4683	2.6250	0.0087	0.0462
Fmnl2	95.0303	-1.0843	0.2959	-3.6641	0.0002	0.0032
Fos	77.1479	3.9226	0.3555	11.0341	0.0000	0.0000
Foxs1	32.0697	1.2815	0.4335	2.9563	0.0031	0.0223
Fst	67.2950	1.1645	0.3551	3.2798	0.0010	0.0098
Furin	30.2879	1.2882	0.4835	2.6641	0.0077	0.0424
Gadd45b	253.1363	1.3640	0.1853	7.3597	0.0000	0.0000
Gadd45g	171.0983	1.3813	0.2158	6.3997	0.0000	0.0000
Gbp2	45.9944	1.0218	0.3913	2.6113	0.0090	0.0476
Gpc4	88.2074	1.1128	0.3127	3.5582	0.0004	0.0045
Gpx4	804.9361	1.0320	0.1531	6.7391	0.0000	0.0000
Grasp	72.3715	1.0373	0.3534	2.9347	0.0033	0.0234
Grid1	34.0780	-1.2859	0.4861	-2.6451	0.0082	0.0442
H1f2	45.0781	2.0650	0.3665	5.6335	0.0000	0.0000
Hbegf	204.8516	2.2831	0.2451	9.3141	0.0000	0.0000
Hdac6	123.4053	1.1568	0.2802	4.1284	0.0000	0.0007
Hes1	144.0883	2.0248	0.2303	8.7917	0.0000	0.0000
Higd1a	194.7448	1.0368	0.2041	5.0810	0.0000	0.0000
Hilpda	25.7586	1.3196	0.4949	2.6663	0.0077	0.0422
Hk2	250.3834	1.0536	0.1849	5.6973	0.0000	0.0000
Hmcn1	73.4503	1.4117	0.3352	4.2117	0.0000	0.0005
Hoxd13	40.3140	1.1444	0.3998	2.8623	0.0042	0.0274
Id3	700.0648	1.1451	0.1586	7.2197	0.0000	0.0000
Ier2	181.5340	2.3085	0.2329	9.9105	0.0000	0.0000
Ier3	172.3556	3.4379	0.2334	14.7274	0.0000	0.0000
Ier5l	71.6539	2.0092	0.3204	6.2720	0.0000	0.0000
Ifnar2	60.3206	1.2032	0.3598	3.3438	0.0008	0.0082
Ift43	60.8711	1.0920	0.3629	3.0093	0.0026	0.0195
Igf2bp3	138.1351	-1.3427	0.3077	-4.3638	0.0000	0.0003
Insm2	43.9080	-1.2039	0.4150	-2.9006	0.0037	0.0253
Irf1	80.2788	2.4415	0.2891	8.4437	0.0000	0.0000
Itgax	25.9840	1.3402	0.4893	2.7391	0.0062	0.0358
Jun	797.1934	1.0272	0.1314	7.8189	0.0000	0.0000
Junb	530.2543	3.0277	0.1574	19.2353	0.0000	0.0000
Klf10	95.9119	1.9703	0.3089	6.3775	0.0000	0.0000
Kxd1	33.3000	1.3488	0.4639	2.9077	0.0036	0.0249
Lama5	174.5593	1.0794	0.2502	4.3143	0.0000	0.0003
Lamc2	149.9568	1.9571	0.6021	3.2503	0.0012	0.0106
Lamtor3	77.4094	1.0529	0.2945	3.5748	0.0004	0.0042
Lats2	131.1826	1.2837	0.2409	5.3276	0.0000	0.0000
Ldlr	80.1064	1.4056	0.3123	4.5004	0.0000	0.0002
Lhfpl2	67.8972	1.0752	0.3286	3.2721	0.0011	0.0100
Lif	40.8099	4.1285	0.4890	8.4435	0.0000	0.0000
LOC100360449	9544.1123	2.2462	0.1955	11.4903	0.0000	0.0000
LOC100360828	94.2951	1.2583	0.2726	4.6160	0.0000	0.0001
LOC100365062	435.6789	1.2921	0.2174	5.9427	0.0000	0.0000
LOC100909595	139.4697	1.2766	0.2536	5.0332	0.0000	0.0000
LOC100909857	41.4320	1.1630	0.4165	2.7921	0.0052	0.0320
LOC100911668	45.8444	1.1666	0.4107	2.8403	0.0045	0.0289
LOC100911727	114.8116	-1.0669	0.3295	-3.2374	0.0012	0.0109

LOC103689931	41.1221	1.5190	0.4598	3.3038	0.0010	0.0092
LOC103691744	30.5508	1.4233	0.4884	2.9142	0.0036	0.0246
LOC103694910	30.4638	-1.8982	0.5711	-3.3236	0.0009	0.0086
LOC108348065	29.3584	-7.7487	1.6704	-4.6388	0.0000	0.0001
LOC108348083	286.3942	-7.8052	1.0336	-7.5512	0.0000	0.0000
LOC108348142	803.5236	-10.7744	2.1066	-5.1146	0.0000	0.0000
LOC108349606	414.5113	1.1148	0.1767	6.3075	0.0000	0.0000
LOC679711	81.5359	1.3588	0.2905	4.6783	0.0000	0.0001
LOC680254	27.4691	1.2797	0.4792	2.6708	0.0076	0.0418
LOC690283	45.7456	-1.1451	0.4113	-2.7839	0.0054	0.0325
LOC691807	142.0082	1.1579	0.2231	5.1904	0.0000	0.0000
Lrp4	58.0522	1.2133	0.3432	3.5353	0.0004	0.0047
Lrrc40	71.2115	1.0899	0.3154	3.4553	0.0005	0.0060
Lsm7	102.5155	1.1116	0.2755	4.0350	0.0001	0.0009
Lsp1	107.9677	1.1395	0.2568	4.4366	0.0000	0.0002
Mafk	189.1548	1.1998	0.2421	4.9562	0.0000	0.0000
Magi1	134.7727	-1.1585	0.2420	-4.7870	0.0000	0.0001
Map2k3	42.7725	1.7011	0.4510	3.7717	0.0002	0.0023
Mdm2	531.9227	1.1610	0.1468	7.9062	0.0000	0.0000
Med28	115.1578	1.0192	0.2438	4.1798	0.0000	0.0006
Mfap5	338.3008	1.1037	0.1598	6.9066	0.0000	0.0000
MGC105649	24.6300	1.7507	0.5284	3.3130	0.0009	0.0089
Mgst2	151.8386	1.2318	0.2307	5.3396	0.0000	0.0000
Mipep	37.2337	1.1029	0.4142	2.6628	0.0077	0.0425
Mlycd	37.2617	1.0545	0.4062	2.5957	0.0094	0.0491
Mmp14	213.0939	1.1117	0.2114	5.2580	0.0000	0.0000
Mrpl14	52.6010	1.0918	0.3687	2.9616	0.0031	0.0220
Mrpl18	72.8277	1.0057	0.3109	3.2351	0.0012	0.0110
Mrpl32	51.2059	1.0763	0.3731	2.8846	0.0039	0.0262
Mrpl33	110.1328	1.5546	0.2528	6.1497	0.0000	0.0000
Mrpl54	126.5500	1.0287	0.2322	4.4305	0.0000	0.0002
Mrps25	93.4372	1.1662	0.2700	4.3199	0.0000	0.0003
Mrps26	79.4375	1.0774	0.2953	3.6489	0.0003	0.0034
Mt-nd2	56.1142	1.3163	0.3900	3.3749	0.0007	0.0075
Mt1	53.8929	2.4942	0.3445	7.2404	0.0000	0.0000
Mtg2	58.9950	1.3525	0.3395	3.9836	0.0001	0.0011
Myadm	43.3854	1.4332	0.4114	3.4836	0.0005	0.0055
Myd88	33.5960	1.2780	0.4528	2.8225	0.0048	0.0301
Nabp1	20.6006	1.6798	0.5688	2.9533	0.0031	0.0224
Nav2	22.4209	1.7047	0.5957	2.8614	0.0042	0.0274
Ncoa2	50.4476	-1.4454	0.4207	-3.4356	0.0006	0.0063
Ndufa13	137.7412	1.0866	0.2542	4.2751	0.0000	0.0004
Ndufa2	78.1806	1.3622	0.3208	4.2468	0.0000	0.0004
Ndufa7	182.9261	1.0830	0.2174	4.9809	0.0000	0.0000
Nedd9	104.0488	1.2048	0.2656	4.5360	0.0000	0.0001
Nefh	95.4935	-1.0368	0.3198	-3.2417	0.0012	0.0108
Nfkbia	633.1983	1.6753	0.1331	12.5871	0.0000	0.0000
Nfkbiz	36.2278	3.3272	0.4449	7.4788	0.0000	0.0000
Npc2	193.0060	1.0280	0.2520	4.0792	0.0000	0.0008
Nptxr	33.4996	1.4193	0.5075	2.7969	0.0052	0.0318
Nr4a1	20.8796	2.5887	0.5833	4.4381	0.0000	0.0002
Nsmce2	96.5043	1.1655	0.2787	4.1823	0.0000	0.0006

Nuak2	65.4544	2.7597	0.3625	7.6132	0.0000	0.0000
Olfml2a	35.5123	1.1549	0.4181	2.7621	0.0057	0.0341
Ophn1	19.3071	2.1682	0.6730	3.2215	0.0013	0.0114
Oxnad1	69.2439	1.1847	0.3019	3.9247	0.0001	0.0014
Paxx	25.2881	1.5575	0.5254	2.9643	0.0030	0.0219
Pcdh9	85.8206	-1.2193	0.3119	-3.9088	0.0001	0.0014
Pdcl3	212.1062	1.0227	0.1938	5.2758	0.0000	0.0000
Pde8a	47.1867	-1.1382	0.4211	-2.7032	0.0069	0.0389
Pdgfc	66.8608	1.2758	0.3430	3.7195	0.0002	0.0027
Pdlim7	53.9972	1.1237	0.3924	2.8638	0.0042	0.0274
Phlda1	50.7148	1.6780	0.4011	4.1840	0.0000	0.0006
Piezo1	66.8194	1.1141	0.3207	3.4741	0.0005	0.0057
Pigg	22.7276	-1.6915	0.5582	-3.0302	0.0024	0.0186
Pih1d1	37.2132	1.1405	0.4161	2.7412	0.0061	0.0356
Pla1a	23.9291	1.7416	0.5379	3.2378	0.0012	0.0109
Plaur	48.1953	1.4131	0.4415	3.2003	0.0014	0.0120
Plcd3	43.0483	1.1223	0.4027	2.7868	0.0053	0.0324
Plekhj1	57.5495	1.1851	0.3391	3.4953	0.0005	0.0053
Plk2	668.2174	1.4629	0.1405	10.4160	0.0000	0.0000
Plscr1	162.3943	1.1612	0.2215	5.2431	0.0000	0.0000
Pmaip1	547.7939	1.4013	0.1579	8.8741	0.0000	0.0000
Podnl1	48.5520	1.2052	0.4210	2.8628	0.0042	0.0274
Ppial4d	88.9952	1.7154	0.3164	5.4215	0.0000	0.0000
Ppidl1	44.2249	1.4325	0.4035	3.5505	0.0004	0.0046
Ppm1e	81.3402	-1.1136	0.3041	-3.6617	0.0003	0.0033
Prmt1	246.1851	1.3680	0.2050	6.6746	0.0000	0.0000
Prrx2	43.0536	1.0382	0.3853	2.6944	0.0071	0.0396
Psmg4	38.6204	1.0479	0.4032	2.5986	0.0094	0.0488
Ptgs2	545.8326	4.6275	0.2153	21.4966	0.0000	0.0000
Ptpn1	53.1624	1.2927	0.3473	3.7226	0.0002	0.0027
Ptrh1	89.4088	1.0603	0.2836	3.7393	0.0002	0.0026
Pvr	215.6905	1.1866	0.2325	5.1030	0.0000	0.0000
Qsox2	60.4458	1.0799	0.3733	2.8930	0.0038	0.0257
Rab20	25.8890	1.7020	0.5079	3.3512	0.0008	0.0080
Ranbp10	70.0450	1.3237	0.3293	4.0191	0.0001	0.0010
Rasl11b	117.8936	1.6308	0.2569	6.3490	0.0000	0.0000
Rgcc	56.9289	1.1735	0.3428	3.4234	0.0006	0.0065
RGD1560324	64.8816	1.1167	0.3432	3.2536	0.0011	0.0106
RGD1562690	185.3911	-10.1889	1.2019	-8.4771	0.0000	0.0000
RGD1566373	28.5759	-1.9883	0.7116	-2.7942	0.0052	0.0320
RGD1597339	75.9922	1.2615	0.3094	4.0773	0.0000	0.0008
Rgs3	116.3603	1.7175	0.2438	7.0456	0.0000	0.0000
Rhob	489.4917	1.1662	0.1676	6.9563	0.0000	0.0000
Ripor1	59.7015	1.0556	0.3790	2.7853	0.0053	0.0325
Rmi1	51.1134	1.1316	0.3740	3.0254	0.0025	0.0187
Rnd1	112.4799	1.4199	0.2562	5.5418	0.0000	0.0000
Rpl8	803.5236	-10.7744	2.1066	-5.1146	0.0000	0.0000
Rpl9	826.5306	3.6813	1.0447	3.5239	0.0004	0.0049
Rps28	736.5198	1.0359	0.1362	7.6043	0.0000	0.0000
Rps3a	2946.0315	1.1468	0.1153	9.9486	0.0000	0.0000
Rrad	221.2356	1.1203	0.2045	5.4780	0.0000	0.0000
RT1-M3-1	24.4164	1.5010	0.5280	2.8430	0.0045	0.0287

Runx2	26.3740	1.3986	0.5041	2.7746	0.0055	0.0331
S1pr3	131.2183	1.0000	0.2355	4.2469	0.0000	0.0004
Satb1	45.8220	-1.2906	0.4145	-3.1132	0.0019	0.0151
Sdc4	508.2893	1.5815	0.1379	11.4662	0.0000	0.0000
Sergef	32.9877	1.1804	0.4534	2.6037	0.0092	0.0484
Serpine1	124.8108	2.0009	0.2873	6.9651	0.0000	0.0000
Setd3	141.7604	1.0023	0.2298	4.3616	0.0000	0.0003
Setd4	63.0983	1.0662	0.3830	2.7838	0.0054	0.0325
Sft2d2	115.5055	1.3876	0.3070	4.5205	0.0000	0.0002
Sgcb	74.0868	1.0884	0.3231	3.3692	0.0008	0.0076
Shank1	19.5234	4.5347	0.9901	4.5801	0.0000	0.0001
Sirt5	68.9738	1.1146	0.3279	3.3994	0.0007	0.0069
Slc1a5	55.9341	1.1055	0.3424	3.2287	0.0012	0.0112
Slc30a1	146.4874	1.1283	0.2364	4.7734	0.0000	0.0001
Slc35e4	48.8737	1.0079	0.3766	2.6763	0.0074	0.0413
Slc44a2	102.2315	1.0020	0.2705	3.7042	0.0002	0.0029
Slc7a5	59.1908	1.7538	0.3640	4.8181	0.0000	0.0000
Smim14	85.3926	1.2607	0.2769	4.5523	0.0000	0.0001
Smim3	125.1611	1.0138	0.2456	4.1278	0.0000	0.0007
Smim8	20.6183	1.6440	0.5931	2.7719	0.0056	0.0333
Sncg	36.1336	1.1986	0.4292	2.7925	0.0052	0.0320
Snx7	115.2808	1.0764	0.2531	4.2536	0.0000	0.0004
Spc25	33.9567	1.1221	0.4293	2.6139	0.0090	0.0473
Sphk1	181.9039	1.0257	0.2183	4.6978	0.0000	0.0001
Sptlc1	80.8459	1.0076	0.2991	3.3690	0.0008	0.0076
Srpx2	70.4627	1.0527	0.3290	3.2000	0.0014	0.0120
St6gal1	22.3312	1.9259	0.5945	3.2396	0.0012	0.0109
Strap	225.7472	1.1404	0.1998	5.7075	0.0000	0.0000
Sub1	230.4498	1.0684	0.1778	6.0091	0.0000	0.0000
Taf15	308.2269	-1.0810	0.2237	-4.8319	0.0000	0.0000
Tcirg1	66.4570	1.2313	0.3110	3.9589	0.0001	0.0012
Tctn1	27.9903	1.5622	0.5089	3.0698	0.0021	0.0169
Tdrp	25.1693	1.4557	0.5304	2.7443	0.0061	0.0354
Tent5a	72.7055	1.2461	0.3682	3.3844	0.0007	0.0073
Tgfb2	104.4113	1.3281	0.2832	4.6894	0.0000	0.0001
Tgfb3	389.2449	1.0515	0.1558	6.7479	0.0000	0.0000
Tgif1	53.0242	1.3633	0.3732	3.6527	0.0003	0.0033
Thbs1	1176.5278	1.0430	0.1228	8.4962	0.0000	0.0000
Thbs2	119.7267	1.2135	0.2344	5.1764	0.0000	0.0000
Timmdc1	48.5205	1.0046	0.3710	2.7075	0.0068	0.0385
Tiparp	249.9302	1.0460	0.1862	5.6180	0.0000	0.0000
Tmbim1	96.1474	1.3141	0.3338	3.9365	0.0001	0.0013
Tmbim6	90.9974	1.0340	0.3268	3.1641	0.0016	0.0132
Tmem199	91.5976	1.0776	0.2915	3.6970	0.0002	0.0029
Tnfaip2	120.2405	1.6054	0.2346	6.8432	0.0000	0.0000
Tob1	651.1517	1.8563	0.1777	10.4462	0.0000	0.0000
Trafd1	116.7693	1.0548	0.2581	4.0867	0.0000	0.0008
Triap1	49.8785	1.0003	0.3596	2.7816	0.0054	0.0326
Trim30c	31.3234	1.6611	0.5251	3.1637	0.0016	0.0133
Trim5	84.5547	1.0198	0.2819	3.6179	0.0003	0.0037
Tsku	80.1543	1.2593	0.3025	4.1624	0.0000	0.0006
Tspan18	39.5670	1.2078	0.4562	2.6476	0.0081	0.0440

Tstd3	39.9061	1.3293	0.4182	3.1784	0.0015	0.0128
Txnip	1039.2442	-1.5605	0.1130	-13.8098	0.0000	0.0000
Tymp	68.0988	1.0802	0.3163	3.4156	0.0006	0.0066
Uba52	1913.9661	1.1864	0.1311	9.0494	0.0000	0.0000
Ube2t	26.9563	1.6238	0.5461	2.9735	0.0029	0.0214
Uqcrb	177.8252	1.1049	0.2212	4.9958	0.0000	0.0000
Uqcrq	180.9077	1.0834	0.2041	5.3082	0.0000	0.0000
Usp18	33.1849	1.2471	0.4458	2.7975	0.0052	0.0317
Vrk2	64.7220	1.1021	0.3312	3.3280	0.0009	0.0085
Wfdc2	25.9529	1.6126	0.5327	3.0273	0.0025	0.0187
Wipf1	28.6396	1.9698	0.5347	3.6837	0.0002	0.0030
Zbtb33	84.3300	-1.0758	0.3888	-2.7668	0.0057	0.0337
Zfp367	1061.5273	-1.2832	0.1336	-9.6063	0.0000	0.0000
Zfp36l1	37.4279	2.8410	0.5048	5.6276	0.0000	0.0000
Zfp36l2	393.9550	1.4876	0.1681	8.8511	0.0000	0.0000
Znrd1	30.9367	1.2831	0.4629	2.7717	0.0056	0.0333
Zyx	122.1477	1.3417	0.2995	4.4801	0.0000	0.0002

Supplementary Table 3B: STRINGdb Input for Isoproterenol vs Basal (Used to generate STRINGdb plots and tables)

	baseMean	log2FC	lfcSE	stat	pvalue	padj
AABR07007121.1	75.5766	1.5527	0.3058	5.0774	0.0000	0.0000
AABR07007905.2	12.7997	2.5511	0.7276	3.5060	0.0005	0.0022
AABR07007997.1	12.6257	4.0723	0.9881	4.1215	0.0000	0.0003
AABR07021220.1	214.8562	-10.2531	2.0167	-5.0842	0.0000	0.0000
AABR07021759.1	4615.8076	-7.0791	0.4004	-17.6787	0.0000	0.0000
AABR07027902.1	25.9953	2.2825	0.6288	3.6298	0.0003	0.0015
AABR07028349.1	395.5070	-5.6627	0.6651	-8.5137	0.0000	0.0000
AABR07030911.1	132.5275	-9.6288	2.5627	-3.7572	0.0002	0.0010
AABR07031089.1	2073.3261	-7.7728	1.0014	-7.7622	0.0000	0.0000
AABR07032255.1	1355.8949	-7.8267	1.6129	-4.8525	0.0000	0.0000
AABR07032904.1	13.9691	2.4453	0.7087	3.4505	0.0006	0.0027
AABR07034639.1	553.5015	1.8251	0.5264	3.4671	0.0005	0.0025
AABR07036855.1	67.7383	1.5422	0.3308	4.6622	0.0000	0.0000
AABR07037203.1	144.9665	-1.5326	0.2791	-5.4914	0.0000	0.0000
AABR07037489.1	36.5149	1.9454	0.5020	3.8756	0.0001	0.0006
AABR07042611.1	43.9641	0.9419	0.3905	2.4124	0.0158	0.0446
AABR07044273.1	349.6165	-6.4718	1.7493	-3.6996	0.0002	0.0012
AABR07044914.1	14.2263	2.5949	0.7473	3.4725	0.0005	0.0025
AABR07049320.1	1450.6426	-1.9861	0.5151	-3.8554	0.0001	0.0007
AABR07049405.1	486.4439	-1.1198	0.1541	-7.2679	0.0000	0.0000
AABR07051190.1	34.0649	-2.3296	0.6207	-3.7533	0.0002	0.0010
AABR07055280.1	270.6606	-24.5505	2.6058	-9.4214	0.0000	0.0000
AABR07055527.1	16.7992	-2.9137	0.7451	-3.9106	0.0001	0.0006
AABR07055826.1	15.9882	2.0523	0.6717	3.0552	0.0022	0.0087
AABR07055919.1	14.6477	2.4596	0.7421	3.3143	0.0009	0.0041
AABR07061825.1	328.4441	-10.7339	1.8168	-5.9082	0.0000	0.0000
AABR07063893.1	12.8829	3.8238	0.9156	4.1761	0.0000	0.0002
AABR07064983.1	44.2763	-1.0353	0.4374	-2.3668	0.0179	0.0492
AABR07064992.1	12.5773	-6.6180	1.4220	-4.6540	0.0000	0.0000
Abhd10	18.6898	4.5140	0.8423	5.3593	0.0000	0.0000
AC106648.1	16.0480	-2.9524	0.9624	-3.0676	0.0022	0.0084
AC107531.3	13.6813	2.6598	0.8175	3.2535	0.0011	0.0049
AC109048.1	57.2222	1.2860	0.3399	3.7831	0.0002	0.0009
AC112624.1	7.8374	3.7083	1.1502	3.2240	0.0013	0.0053
AC141489.1	836.8527	1.4425	0.1373	10.5089	0.0000	0.0000
Ackr3	271.0893	0.8039	0.1772	4.5370	0.0000	0.0000
Actg1	294.7604	-30.0000	4.7123	-6.3663	0.0000	0.0000
Actg2	480.5310	0.8567	0.1868	4.5859	0.0000	0.0000
Acyp1	55.0920	1.7279	0.3641	4.7459	0.0000	0.0000
Adgre5	21.3109	2.0889	0.6483	3.2222	0.0013	0.0054
Adora1	19.4756	-1.6391	0.6647	-2.4659	0.0137	0.0395
Agpat2	29.1127	1.2157	0.4912	2.4747	0.0133	0.0387
Agrn	26.1091	1.2234	0.5021	2.4365	0.0148	0.0422
Akr1c12	32.5490	1.5116	0.4340	3.4828	0.0005	0.0024
Aldh18a1	295.5728	-3.8902	0.5221	-7.4506	0.0000	0.0000
Angpt4	55.1176	0.8658	0.3516	2.4627	0.0138	0.0397
Ankrd1	1940.0372	0.9813	0.0963	10.1894	0.0000	0.0000
Arhgef5	32.0314	1.5983	0.4610	3.4667	0.0005	0.0025

Arrb2	14.6815	-2.1517	0.7778	-2.7663	0.0057	0.0189
Arrdc3	590.8087	-0.5048	0.1696	-2.9769	0.0029	0.0108
Arrdc4	10.5118	-2.7332	1.1376	-2.4025	0.0163	0.0456
Asb5	138.7669	1.7901	0.2351	7.6131	0.0000	0.0000
Ascl1	28.4023	-2.6353	0.5955	-4.4256	0.0000	0.0001
Asic1	32.8682	-1.1445	0.4794	-2.3873	0.0170	0.0472
Atp5md	468.2547	1.6016	0.1760	9.0977	0.0000	0.0000
Atpsckmt	29.3010	1.4438	0.4560	3.1665	0.0015	0.0063
Aven	43.1469	1.0793	0.3913	2.7584	0.0058	0.0193
B4gat1	129.8906	1.3393	0.2305	5.8111	0.0000	0.0000
Bag5l1	51.6729	1.3678	0.4096	3.3398	0.0008	0.0038
Bcar1	102.2341	1.7929	0.3110	5.7641	0.0000	0.0000
Bdkrb2	18.3850	2.1287	0.6117	3.4800	0.0005	0.0024
Bhlhe40	55.3795	1.4877	0.4007	3.7126	0.0002	0.0011
Borcs8	46.4438	1.5641	0.3983	3.9273	0.0001	0.0005
Btf3l4	116.3502	1.3099	0.2675	4.8963	0.0000	0.0000
Btg2	177.1339	1.1231	0.2492	4.5065	0.0000	0.0001
C1s	173.0811	0.9700	0.2022	4.7982	0.0000	0.0000
Cacna2d4	27.9175	1.2919	0.4705	2.7457	0.0060	0.0200
Casp12	72.5978	1.2646	0.3344	3.7822	0.0002	0.0009
Casp8	25.1296	1.9729	0.5350	3.6877	0.0002	0.0012
Ccdc115	51.1331	1.2448	0.3602	3.4558	0.0005	0.0026
Ccdc12	25.7199	1.7246	0.5079	3.3956	0.0007	0.0032
Ccdc126	71.4178	1.7763	0.3222	5.5130	0.0000	0.0000
Ccdc71l	47.5805	-1.2581	0.4218	-2.9831	0.0029	0.0106
Ccdc97	35.1480	1.4176	0.4244	3.3401	0.0008	0.0038
Ccn1	743.1021	0.7631	0.1943	3.9274	0.0001	0.0005
Ccn2	999.2445	0.6618	0.1740	3.8038	0.0001	0.0008
Cdkn2a	95.3873	1.1163	0.2822	3.9562	0.0001	0.0005
Cdkn2b	55.0758	0.9781	0.3350	2.9198	0.0035	0.0127
Cenpw	27.8249	1.8868	0.4950	3.8114	0.0001	0.0008
Cers4	30.2469	1.8451	0.5155	3.5789	0.0003	0.0018
Cfap58l1	7.2409	2.6366	1.1163	2.3618	0.0182	0.0497
Chac1	152.2729	-1.4323	0.2901	-4.9382	0.0000	0.0000
Clcf1	53.3726	1.1036	0.3781	2.9186	0.0035	0.0127
Clec2d	26.8828	1.3792	0.4955	2.7836	0.0054	0.0182
Clec2g	231.7265	1.0307	0.1829	5.6360	0.0000	0.0000
Coa3	43.4308	0.9282	0.3848	2.4123	0.0159	0.0446
Col12a1	50.6316	0.9050	0.3678	2.4604	0.0139	0.0399
Col3a1	6379.7175	1.5083	0.1014	14.8787	0.0000	0.0000
Comt	26.2714	2.1424	0.5861	3.6556	0.0003	0.0014
Cox5b	346.0402	1.1146	0.1893	5.8887	0.0000	0.0000
Cox7a2l2	275.5358	1.5924	0.1868	8.5246	0.0000	0.0000
Cox7b	436.2847	1.4577	0.1572	9.2718	0.0000	0.0000
Cox7b2	26.0083	1.7865	0.5123	3.4870	0.0005	0.0024
Cpg1	11.1309	2.0535	0.7599	2.7022	0.0069	0.0223
Cr1l	164.8916	1.2598	0.2158	5.8391	0.0000	0.0000
Creld2	94.9288	1.2522	0.2868	4.3660	0.0000	0.0001
Csrnp1	83.0179	1.2162	0.3356	3.6240	0.0003	0.0015
Cxadr	21.1075	1.2504	0.5219	2.3956	0.0166	0.0463
D2hgdh	33.9924	1.9745	0.4631	4.2633	0.0000	0.0001
Daglb	42.8348	1.4377	0.4248	3.3847	0.0007	0.0033

Dap	52.4312	1.0581	0.3965	2.6687	0.0076	0.0242
Decr1	71.4964	1.4342	0.3140	4.5673	0.0000	0.0000
Def6	35.1493	1.0854	0.4067	2.6692	0.0076	0.0242
Dhrs7b	57.2071	1.5889	0.3406	4.6655	0.0000	0.0000
Dhtkd1	12.8468	2.1619	0.8492	2.5457	0.0109	0.0328
Dkk4	14.1669	1.8541	0.7184	2.5809	0.0099	0.0301
Dnajc24	24.1183	1.7513	0.5264	3.3272	0.0009	0.0039
Dock8	39.1149	1.0298	0.4355	2.3646	0.0180	0.0494
Dok5	18.8809	1.8336	0.5848	3.1354	0.0017	0.0069
Dpcd	51.7870	1.8660	0.3839	4.8609	0.0000	0.0000
Dram1	101.3773	1.0069	0.2807	3.5873	0.0003	0.0017
Duoxa1	30.8566	1.3205	0.4512	2.9264	0.0034	0.0125
Egr1	160.3659	1.3876	0.3978	3.4886	0.0005	0.0024
Epha2	117.8874	1.4395	0.2767	5.2029	0.0000	0.0000
Eps8	29.5403	1.4657	0.5139	2.8520	0.0043	0.0152
Erbb2	16.1988	1.9183	0.7020	2.7326	0.0063	0.0206
Ercc5	26.3525	1.6231	0.5849	2.7749	0.0055	0.0185
Ermard	28.1303	1.7285	0.4680	3.6935	0.0002	0.0012
Errfi1	268.0153	0.5532	0.1993	2.7751	0.0055	0.0185
Esco2	39.8813	2.0670	0.4152	4.9780	0.0000	0.0000
Evc	43.6594	1.6630	0.3989	4.1690	0.0000	0.0002
Evc2	54.1346	1.3477	0.3463	3.8913	0.0001	0.0006
Exosc4	76.2290	1.3332	0.2999	4.4458	0.0000	0.0001
F3	194.1116	1.8616	0.2479	7.5092	0.0000	0.0000
Faap100	11.2862	2.2395	0.8217	2.7253	0.0064	0.0210
Fbf1	24.8878	1.7056	0.5103	3.3424	0.0008	0.0037
Fcgrt	429.2497	1.3220	0.1561	8.4670	0.0000	0.0000
Fgg	14.8226	1.6386	0.6345	2.5822	0.0098	0.0301
Filip1	14.9389	2.0558	0.6861	2.9963	0.0027	0.0103
Fktn	34.8600	1.2180	0.4658	2.6151	0.0089	0.0277
Flrt3	76.6740	-1.5558	0.3202	-4.8587	0.0000	0.0000
Flt1	33.2039	1.4472	0.4604	3.1431	0.0017	0.0068
Fmnl2	95.0303	-1.0184	0.2894	-3.5186	0.0004	0.0021
Foxj2	21.3305	1.4899	0.5540	2.6895	0.0072	0.0230
Fst	67.2950	1.0007	0.3539	2.8275	0.0047	0.0162
Gadd45b	253.1363	1.5768	0.1833	8.6001	0.0000	0.0000
Gadd45g	171.0983	1.4421	0.2141	6.7355	0.0000	0.0000
Gbp2	45.9944	1.4764	0.3817	3.8676	0.0001	0.0007
Gpat3	22.6216	1.5017	0.5284	2.8420	0.0045	0.0156
Gpc4	88.2074	1.3013	0.3087	4.2149	0.0000	0.0002
Gpx4	804.9361	1.3298	0.1521	8.7420	0.0000	0.0000
Grasp	72.3715	1.1092	0.3500	3.1693	0.0015	0.0063
Grid1	34.0780	-1.3956	0.4800	-2.9077	0.0036	0.0131
Gstz1	20.1536	1.7282	0.5716	3.0235	0.0025	0.0095
Gxylt1	47.0203	1.3262	0.4243	3.1253	0.0018	0.0071
Hbegf	204.8516	1.0003	0.2529	3.9557	0.0001	0.0005
Hdac6	123.4053	1.4226	0.2755	5.1641	0.0000	0.0000
Hdhd5	20.4599	1.6184	0.5471	2.9581	0.0031	0.0114
Hes1	144.0883	0.6724	0.2382	2.8229	0.0048	0.0164
Hgd	12.6025	1.9873	0.7540	2.6359	0.0084	0.0263
Higd1a	194.7448	1.2137	0.2015	6.0224	0.0000	0.0000
Hk2	250.3834	1.0514	0.1835	5.7302	0.0000	0.0000

Hmcn1	73.4503	1.5178	0.3319	4.5724	0.0000	0.0000
Hmmr	31.8192	1.6256	0.4618	3.5202	0.0004	0.0021
Id3	700.0648	1.3341	0.1577	8.4591	0.0000	0.0000
Ier2	181.5340	1.2004	0.2398	5.0060	0.0000	0.0000
Ier3	172.3556	1.1515	0.2476	4.6501	0.0000	0.0000
Ifnar2	60.3206	1.4611	0.3538	4.1291	0.0000	0.0002
Ift43	60.8711	1.5763	0.3541	4.4511	0.0000	0.0001
Igf2bp2	22.5069	1.3263	0.5165	2.5677	0.0102	0.0311
Igf2bp3	138.1351	-1.7670	0.3105	-5.6902	0.0000	0.0000
Ikbkg	27.2170	1.8370	0.4930	3.7260	0.0002	0.0011
Il15ra	7.3744	4.0942	1.5037	2.7228	0.0065	0.0212
Il6	13.6836	2.7121	0.8197	3.3086	0.0009	0.0041
Inhbe	11.2866	1.8933	0.7969	2.3757	0.0175	0.0483
Insm2	43.9080	-1.2860	0.4081	-3.1512	0.0016	0.0066
Irf1	80.2788	0.8194	0.3006	2.7260	0.0064	0.0210
Junb	530.2543	0.8348	0.1646	5.0715	0.0000	0.0000
Kif26a	16.0037	-2.2525	0.7287	-3.0913	0.0020	0.0079
Klf16	18.5924	2.1911	0.6437	3.4039	0.0007	0.0031
Kxd1	33.3000	1.1912	0.4624	2.5764	0.0100	0.0304
Lama5	174.5593	1.2018	0.2480	4.8464	0.0000	0.0000
Lamc2	149.9568	2.1945	0.6010	3.6516	0.0003	0.0014
Lamtor3	77.4094	1.3656	0.2882	4.7390	0.0000	0.0000
Lats2	131.1826	1.3116	0.2386	5.4967	0.0000	0.0000
Ldhd	19.6213	1.5842	0.5647	2.8052	0.0050	0.0172
Ldlr	80.1064	0.9859	0.3139	3.1408	0.0017	0.0068
Lexm	6.6677	2.5851	1.0076	2.5656	0.0103	0.0313
Lhfpl2	67.8972	1.3716	0.3217	4.2629	0.0000	0.0001
LOC100360449	9544.1123	1.5388	0.1955	7.8692	0.0000	0.0000
LOC100360828	94.2951	1.3411	0.2696	4.9747	0.0000	0.0000
LOC100365062	435.6789	1.3551	0.2167	6.2544	0.0000	0.0000
LOC100366030	12.5124	1.9654	0.7567	2.5972	0.0094	0.0290
LOC100909595	139.4697	1.9362	0.2474	7.8263	0.0000	0.0000
LOC100911256	9.9770	3.0478	1.1385	2.6770	0.0074	0.0238
LOC100911456	25.6508	1.5994	0.5517	2.8990	0.0037	0.0134
LOC100911668	45.8444	2.1641	0.3931	5.5053	0.0000	0.0000
LOC100911727	114.8116	-1.7401	0.3376	-5.1548	0.0000	0.0000
LOC100912481	1274.4850	-4.4133	1.7788	-2.4810	0.0131	0.0381
LOC102555217	8.2141	2.0646	0.8473	2.4367	0.0148	0.0422
LOC103689927	22.2413	2.0475	0.7026	2.9141	0.0036	0.0129
LOC103689931	41.1221	1.3868	0.4581	3.0270	0.0025	0.0094
LOC103691556	24.1629	-1.4074	0.5667	-2.4836	0.0130	0.0379
LOC103692716	2395.7205	-27.2125	2.6121	-10.4181	0.0000	0.0000
LOC103693776	15.8883	-2.4925	0.8063	-3.0912	0.0020	0.0079
LOC103694910	30.4638	-5.3540	1.1213	-4.7748	0.0000	0.0000
LOC108348065	29.3584	-4.6135	1.3274	-3.4755	0.0005	0.0025
LOC108348083	286.3942	1.2049	0.1704	7.0728	0.0000	0.0000
LOC108348142	803.5236	-6.4013	1.7896	-3.5769	0.0003	0.0018
LOC108349606	414.5113	1.3602	0.1752	7.7639	0.0000	0.0000
LOC360919	22.5618	1.7308	0.5369	3.2236	0.0013	0.0053
LOC497940	13.6672	1.9139	0.6661	2.8732	0.0041	0.0144
LOC498759	17.0528	2.0737	0.6220	3.3341	0.0009	0.0038
LOC679711	81.5359	1.2590	0.2889	4.3581	0.0000	0.0001

LOC680254	27.4691	1.1728	0.4765	2.4612	0.0138	0.0399
LOC690276	12.9390	1.8755	0.6646	2.8222	0.0048	0.0165
LOC691807	142.0082	1.5077	0.2187	6.8939	0.0000	0.0000
LOC691995	10.3007	-3.0124	0.9445	-3.1894	0.0014	0.0059
Lrp3	16.1877	-2.7522	0.7731	-3.5599	0.0004	0.0019
Lrp4	58.0522	1.1583	0.3405	3.4016	0.0007	0.0031
Lrrc40	71.2115	1.6806	0.3068	5.4769	0.0000	0.0000
Lrrn1	7.8647	-3.2692	1.2994	-2.5160	0.0119	0.0352
Lsm7	102.5155	1.0416	0.2739	3.8027	0.0001	0.0008
Lsp1	107.9677	1.3304	0.2530	5.2580	0.0000	0.0000
Ly6k	18.8961	1.5388	0.5555	2.7701	0.0056	0.0187
Mafk	189.1548	1.6317	0.2383	6.8485	0.0000	0.0000
Magea4	20.4795	1.5658	0.5813	2.6937	0.0071	0.0227
Magi1	134.7727	-0.5969	0.2270	-2.6297	0.0085	0.0267
Mboat7l1	18.8115	1.9490	0.6124	3.1824	0.0015	0.0060
Mdm2	531.9227	1.4874	0.1449	10.2616	0.0000	0.0000
Med28	115.1578	1.3710	0.2386	5.7451	0.0000	0.0000
Meis3	33.1640	-2.1729	0.5545	-3.9186	0.0001	0.0005
Metrn	34.7938	1.1882	0.4610	2.5773	0.0100	0.0304
Mfap5	338.3008	0.9496	0.1593	5.9606	0.0000	0.0000
MGC105649	24.6300	1.5103	0.5281	2.8599	0.0042	0.0149
MGC94199	29.0205	1.2818	0.4532	2.8284	0.0047	0.0162
Mgst2	151.8386	1.5451	0.2270	6.8069	0.0000	0.0000
Mipep	37.2337	1.3963	0.4060	3.4395	0.0006	0.0027
Mlycd	37.2617	0.9687	0.4038	2.3990	0.0164	0.0460
Mmp14	213.0939	0.7109	0.2122	3.3500	0.0008	0.0037
Mrm1	24.4466	2.0523	0.5709	3.5947	0.0003	0.0017
Mrpl14	52.6010	0.9597	0.3669	2.6154	0.0089	0.0277
Mrpl18	72.8277	1.3749	0.3042	4.5192	0.0000	0.0001
Mrpl2	29.5949	1.6512	0.4690	3.5211	0.0004	0.0021
Mrpl32	51.2059	1.3717	0.3662	3.7460	0.0002	0.0010
Mrpl33	110.1328	1.8124	0.2491	7.2760	0.0000	0.0000
Mrpl54	126.5500	1.0162	0.2302	4.4137	0.0000	0.0001
Mrps25	93.4372	1.4858	0.2648	5.6119	0.0000	0.0000
Mrps26	79.4375	1.1724	0.2917	4.0186	0.0001	0.0004
Mtg2	58.9950	1.6602	0.3333	4.9809	0.0000	0.0000
Myadm	43.3854	1.1978	0.4110	2.9140	0.0036	0.0129
Myd88	33.5960	1.2557	0.4489	2.7972	0.0052	0.0175
Nabp1	20.6006	1.4428	0.5684	2.5385	0.0111	0.0334
Ncoa2	50.4476	-1.1637	0.4025	-2.8908	0.0038	0.0137
Ndufa13	137.7412	1.1637	0.2519	4.6202	0.0000	0.0000
Ndufa2	78.1806	1.3987	0.3181	4.3970	0.0000	0.0001
Ndufa7	182.9261	1.3238	0.2144	6.1734	0.0000	0.0000
Nedd9	104.0488	0.9134	0.2659	3.4349	0.0006	0.0028
Nefh	95.4935	-0.7500	0.3065	-2.4469	0.0144	0.0412
NEWGENE_1559832	36.3521	2.9326	1.2265	2.3911	0.0168	0.0468
Nipal2	26.2944	1.9019	0.5140	3.7001	0.0002	0.0012
Nme4	16.1612	1.6310	0.6230	2.6181	0.0088	0.0275
Nnmt	30.1793	1.6561	0.4860	3.4074	0.0007	0.0030
Nptxr	33.4996	1.3728	0.5044	2.7216	0.0065	0.0212
Nsmce2	96.5043	1.7616	0.2713	6.4933	0.0000	0.0000
Nsun6	23.1643	1.7337	0.5444	3.1847	0.0014	0.0060

Nuak2	65.4544	1.3001	0.3743	3.4730	0.0005	0.0025
Nudt7	17.7234	1.4915	0.6262	2.3819	0.0172	0.0477
Nudt9	36.4100	1.3376	0.4361	3.0669	0.0022	0.0084
Olfml2a	35.5123	1.0239	0.4162	2.4602	0.0139	0.0400
Ophn1	19.3071	1.7314	0.6762	2.5605	0.0105	0.0316
Oxnad1	69.2439	1.1310	0.2996	3.7756	0.0002	0.0009
Pacrgl	26.1522	1.3353	0.5109	2.6139	0.0090	0.0278
Paxx	25.2881	1.8392	0.5162	3.5633	0.0004	0.0018
Pcdh9	85.8206	-0.9333	0.2980	-3.1322	0.0017	0.0070
Pdcl3	212.1062	1.3188	0.1903	6.9300	0.0000	0.0000
Pdgfc	66.8608	1.4641	0.3384	4.3261	0.0000	0.0001
Piezo1	66.8194	1.0741	0.3179	3.3786	0.0007	0.0033
Pigy	15.1894	1.5993	0.6235	2.5651	0.0103	0.0313
Pih1d1	37.2132	1.5337	0.4065	3.7729	0.0002	0.0009
Pla1a	23.9291	1.4554	0.5384	2.7031	0.0069	0.0223
Plaur	48.1953	1.4428	0.4374	3.2987	0.0010	0.0043
Plcd3	43.0483	1.4449	0.3940	3.6672	0.0002	0.0013
Plekhj1	57.5495	1.1074	0.3369	3.2869	0.0010	0.0044
Plk2	668.2174	0.7537	0.1420	5.3065	0.0000	0.0000
Plscr1	162.3943	1.6586	0.2164	7.6636	0.0000	0.0000
Plscr4	20.2038	1.3205	0.5567	2.3720	0.0177	0.0487
Pmaip1	547.7939	1.4470	0.1571	9.2132	0.0000	0.0000
Podnl1	48.5520	2.0032	0.4091	4.8964	0.0000	0.0000
Ppial4d	88.9952	0.8968	0.3214	2.7904	0.0053	0.0179
Ppidl1	44.2249	1.6210	0.3973	4.0799	0.0000	0.0003
Ppm1e	81.3402	-0.7697	0.2902	-2.6520	0.0080	0.0252
Ppp1r15a	49.8000	1.5677	0.4166	3.7626	0.0002	0.0009
Prmt1	246.1851	1.6666	0.2026	8.2277	0.0000	0.0000
Procr	15.6542	1.9055	0.6958	2.7386	0.0062	0.0204
Prss36	17.6980	1.5500	0.6213	2.4945	0.0126	0.0369
Psmg4	38.6204	1.3820	0.3949	3.4993	0.0005	0.0023
Ptgs2	545.8326	1.7794	0.2264	7.8609	0.0000	0.0000
Ptpn1	53.1624	1.5341	0.3418	4.4878	0.0000	0.0001
Ptrh1	89.4088	1.3219	0.2785	4.7457	0.0000	0.0000
Pus10	36.0829	1.6012	0.4194	3.8178	0.0001	0.0008
Pvr	215.6905	1.7617	0.2278	7.7318	0.0000	0.0000
Ranbp10	70.0450	1.6431	0.3229	5.0885	0.0000	0.0000
Rasl11b	117.8936	1.2558	0.2578	4.8707	0.0000	0.0000
Rassf9	43.1842	2.1949	0.3961	5.5413	0.0000	0.0000
Rbks	15.3575	1.8921	0.6629	2.8543	0.0043	0.0152
Rgcc	56.9289	1.1691	0.3400	3.4387	0.0006	0.0028
RGD1304624	7.6196	2.6619	1.0402	2.5590	0.0105	0.0317
RGD1309534	33.2153	1.2667	0.4739	2.6727	0.0075	0.0240
RGD1560324	64.8816	1.8889	0.3326	5.6785	0.0000	0.0000
RGD1562690	185.3911	-10.4463	1.2019	-8.6912	0.0000	0.0000
RGD1566373	28.5759	-2.7557	0.7714	-3.5722	0.0004	0.0018
RGD1597339	75.9922	1.7104	0.3027	5.6509	0.0000	0.0000
Rgs3	116.3603	1.4138	0.2441	5.7927	0.0000	0.0000
Rmi1	51.1134	1.3593	0.3678	3.6963	0.0002	0.0012
Rnd1	112.4799	1.0546	0.2571	4.1019	0.0000	0.0003
Rpain	11.1620	2.8927	0.8068	3.5853	0.0003	0.0017
Rpl8	803.5236	-6.4013	1.7896	-3.5769	0.0003	0.0018

Rpl9	826.5306	2.7721	1.0448	2.6532	0.0080	0.0252
Rps28	736.5198	1.2356	0.1352	9.1411	0.0000	0.0000
Rps3a	2946.0315	1.5886	0.1148	13.8345	0.0000	0.0000
Rrad	221.2356	1.3483	0.2020	6.6747	0.0000	0.0000
RT1-CE5	27.5641	1.1851	0.4949	2.3945	0.0166	0.0464
RT1-M3-1	24.4164	1.3835	0.5256	2.6322	0.0085	0.0266
Satb1	45.8220	-2.2416	0.4500	-4.9816	0.0000	0.0000
Sccpdh	83.3944	10.0967	1.2239	8.2498	0.0000	0.0000
Sdc4	508.2893	1.0404	0.1388	7.4928	0.0000	0.0000
Sdr9c7	12.5572	2.1897	0.7873	2.7813	0.0054	0.0183
Selenop	44.4983	1.4317	0.3898	3.6732	0.0002	0.0013
Septin1	33.8797	1.1093	0.4255	2.6070	0.0091	0.0283
Sergef	32.9877	1.7251	0.4409	3.9126	0.0001	0.0006
Serpinb7	11.4207	1.9386	0.7889	2.4572	0.0140	0.0402
Serpine1	124.8108	1.1993	0.2914	4.1150	0.0000	0.0003
Setd3	141.7604	1.4639	0.2247	6.5163	0.0000	0.0000
Setd4	63.0983	1.8130	0.3736	4.8532	0.0000	0.0000
Sft2d2	115.5055	1.4459	0.3049	4.7422	0.0000	0.0000
Sgcb	74.0868	1.1640	0.3194	3.6446	0.0003	0.0014
Sirt5	68.9738	1.3218	0.3229	4.0936	0.0000	0.0003
Ska1	18.7396	1.8446	0.5731	3.2186	0.0013	0.0054
Slc15a1	11.1343	2.1519	0.7745	2.7784	0.0055	0.0184
Slc1a5	55.9341	0.9313	0.3413	2.7290	0.0064	0.0209
Slc25a51	12.1622	2.7349	0.8976	3.0470	0.0023	0.0089
Slc30a1	146.4874	1.9912	0.2299	8.6630	0.0000	0.0000
Slc35e4	48.8737	1.5786	0.3658	4.3159	0.0000	0.0001
Slc44a2	102.2315	0.7201	0.2707	2.6601	0.0078	0.0247
Slc52a2	21.4685	1.4445	0.5520	2.6167	0.0089	0.0276
Slc7a5	59.1908	0.9755	0.3708	2.6307	0.0085	0.0267
Slitrk6	18.3129	-1.7116	0.6632	-2.5810	0.0099	0.0301
Smim14	85.3926	1.3656	0.2734	4.9950	0.0000	0.0000
Smim3	125.1611	1.1445	0.2427	4.7166	0.0000	0.0000
Smim8	20.6183	1.8895	0.5847	3.2318	0.0012	0.0052
Sncg	36.1336	1.2848	0.4237	3.0324	0.0024	0.0093
Snx7	115.2808	1.4931	0.2477	6.0278	0.0000	0.0000
Spc25	33.9567	1.3155	0.4226	3.1125	0.0019	0.0074
Sphk1	181.9039	1.4009	0.2144	6.5348	0.0000	0.0000
Sptlc1	80.8459	1.3183	0.2930	4.4993	0.0000	0.0001
Srpx2	70.4627	1.4016	0.3229	4.3411	0.0000	0.0001
St6gal1	22.3312	1.4846	0.5979	2.4829	0.0130	0.0379
Stoml1	26.1349	1.5872	0.5266	3.0142	0.0026	0.0097
Strap	225.7472	1.1731	0.1982	5.9178	0.0000	0.0000
Sub1	230.4498	1.4761	0.1744	8.4624	0.0000	0.0000
Supt6h	179.1777	-1.9683	0.4681	-4.2045	0.0000	0.0002
Taf15	308.2269	-1.6604	0.2268	-7.3208	0.0000	0.0000
Tcaf2	9.7567	1.9095	0.7781	2.4542	0.0141	0.0405
Tcaim	10.6839	1.8793	0.7564	2.4844	0.0130	0.0378
Tcirg1	66.4570	1.3843	0.3064	4.5186	0.0000	0.0001
Tctn1	27.9903	1.9664	0.4984	3.9456	0.0001	0.0005
Tdrp	25.1693	1.7713	0.5196	3.4088	0.0007	0.0030
Tec	24.8640	1.3565	0.5199	2.6093	0.0091	0.0281
Tex19.2	6.3647	-2.9273	1.2153	-2.4086	0.0160	0.0450

Tgfb2	104.4113	1.3862	0.2807	4.9384	0.0000	0.0000
Tgfb3	389.2449	1.0770	0.1546	6.9653	0.0000	0.0000
Tgfbr3	16.3752	1.6896	0.6994	2.4158	0.0157	0.0443
Thbs1	1176.5278	0.4526	0.1236	3.6608	0.0003	0.0013
Thbs2	119.7267	0.7528	0.2361	3.1888	0.0014	0.0059
Timmdc1	48.5205	1.6506	0.3598	4.5879	0.0000	0.0000
Tiparp	249.9302	0.8777	0.1857	4.7270	0.0000	0.0000
Tipin	36.9493	1.2327	0.4838	2.5481	0.0108	0.0326
Tlcd1	32.8626	1.4979	0.4621	3.2411	0.0012	0.0051
Tlr6	15.1983	2.1793	0.7053	3.0898	0.0020	0.0079
Tmbim1	96.1474	1.4189	0.3308	4.2891	0.0000	0.0001
Tmbim6	90.9974	1.1618	0.3231	3.5963	0.0003	0.0017
Tmem159	13.7862	2.5038	0.8023	3.1209	0.0018	0.0072
Tmem184b	42.5687	1.0205	0.3947	2.5859	0.0097	0.0298
Tmem199	91.5976	1.2398	0.2868	4.3222	0.0000	0.0001
Tmem222	31.1047	-2.2838	0.6426	-3.5540	0.0004	0.0019
Tmem70	23.6517	1.5979	0.5595	2.8558	0.0043	0.0151
Tmsb15b2	17.8388	1.6616	0.5862	2.8347	0.0046	0.0159
Tnfaip2	120.2405	0.8826	0.2382	3.7052	0.0002	0.0011
Tnfaip6	19.1793	1.6550	0.5943	2.7849	0.0054	0.0181
Tnn	18.4406	1.5984	0.6719	2.3787	0.0174	0.0480
Tor4a	32.1948	1.5480	0.4898	3.1608	0.0016	0.0064
Trafd1	116.7693	1.6181	0.2516	6.4323	0.0000	0.0000
Triap1	49.8785	1.2561	0.3530	3.5585	0.0004	0.0019
Trim30c	31.3234	2.4210	0.5109	4.7386	0.0000	0.0000
Trim5	84.5547	1.5127	0.2746	5.5093	0.0000	0.0000
Trim63	29.6227	1.1723	0.4552	2.5755	0.0100	0.0305
Tsku	80.1543	0.9101	0.3035	2.9983	0.0027	0.0102
Tspan18	39.5670	1.6162	0.4456	3.6269	0.0003	0.0015
Tstd3	39.9061	1.6443	0.4110	4.0010	0.0001	0.0004
Txnip	1039.2442	-1.6194	0.1118	-14.4822	0.0000	0.0000
Tymp	68.0988	0.9817	0.3143	3.1234	0.0018	0.0072
Uba52	1913.9661	0.7358	0.1313	5.6041	0.0000	0.0000
Ube2t	26.9563	1.6286	0.5415	3.0074	0.0026	0.0100
Ubl5	36.7692	1.5880	0.4324	3.6722	0.0002	0.0013
Unc5a	17.9077	-2.1465	0.6581	-3.2618	0.0011	0.0048
Uqcrb	177.8252	1.6694	0.2160	7.7272	0.0000	0.0000
Uqcrq	180.9077	1.3825	0.2005	6.8944	0.0000	0.0000
Usb1	21.4348	1.3066	0.5439	2.4023	0.0163	0.0456
Usp18	33.1849	1.1843	0.4425	2.6766	0.0074	0.0238
Vamp5	28.3537	1.3997	0.4878	2.8693	0.0041	0.0146
Vrk2	64.7220	1.6041	0.3227	4.9709	0.0000	0.0000
Vxn	12.1396	2.3362	0.7885	2.9630	0.0030	0.0113
Wdr54	11.7423	2.0747	0.7440	2.7887	0.0053	0.0180
Wfdc2	25.9529	2.1027	0.5207	4.0384	0.0001	0.0003
Wipf1	28.6396	1.8368	0.5328	3.4473	0.0006	0.0027
Zc3h8	38.4374	1.6727	0.3845	4.3504	0.0000	0.0001
Zfand1	11.1307	2.6488	0.8270	3.2031	0.0014	0.0057
Zfp17	23.2962	1.9303	0.5037	3.8320	0.0001	0.0007
Zfp444	32.0768	1.1537	0.4674	2.4684	0.0136	0.0392
Zfp46	9.8738	2.0051	0.8486	2.3629	0.0181	0.0496
Zfyve21	27.0175	1.4236	0.4942	2.8808	0.0040	0.0141

Znrd1	30.9367	1.3408	0.4578	2.9292	0.0034	0.0124

Supplementary Figure 6. Immunostaining of myocarditis biopsies. Serial sections of each myocarditis heart biopsy tissue specimen from Mayo Clinic Pathology were tested in immunohistochemical staining. Fibrosis staining of collagen by Trichrome Stain (**A:** bluish color) was observed in myocarditis heart tissue. IgG staining (**B**, pink/red color) was observed in myocarditis heart tissues and the PBS controls reacted with alkaline phosphatase–conjugated secondary antibody were negative for IgG (**C**). Healthy human myocardium (**D**) was also negative for IgG staining. Mean fibrosis scores (**E**) for IL-17A+ biopsies (n=11) and IL-17A-negative (n=11) biopsies were determined by trichrome staining. (Mann-Whitney p=0.0175). Fibrosis scores were determined using trichrome staining (graded +0.25 to +3). A +4 trichrome score example of severe replacement fibrosis is seen in **PT5** and an example of +3 trichrome score for moderate to marked interstitial fibrosis is seen in **PT1.** Total magnification ×200. (**PT1** = Patient 1, **PT2** = Patient 2, **PT3** = Patient 3, **PT4** = Patient 4, **PT5** = Patient 5)

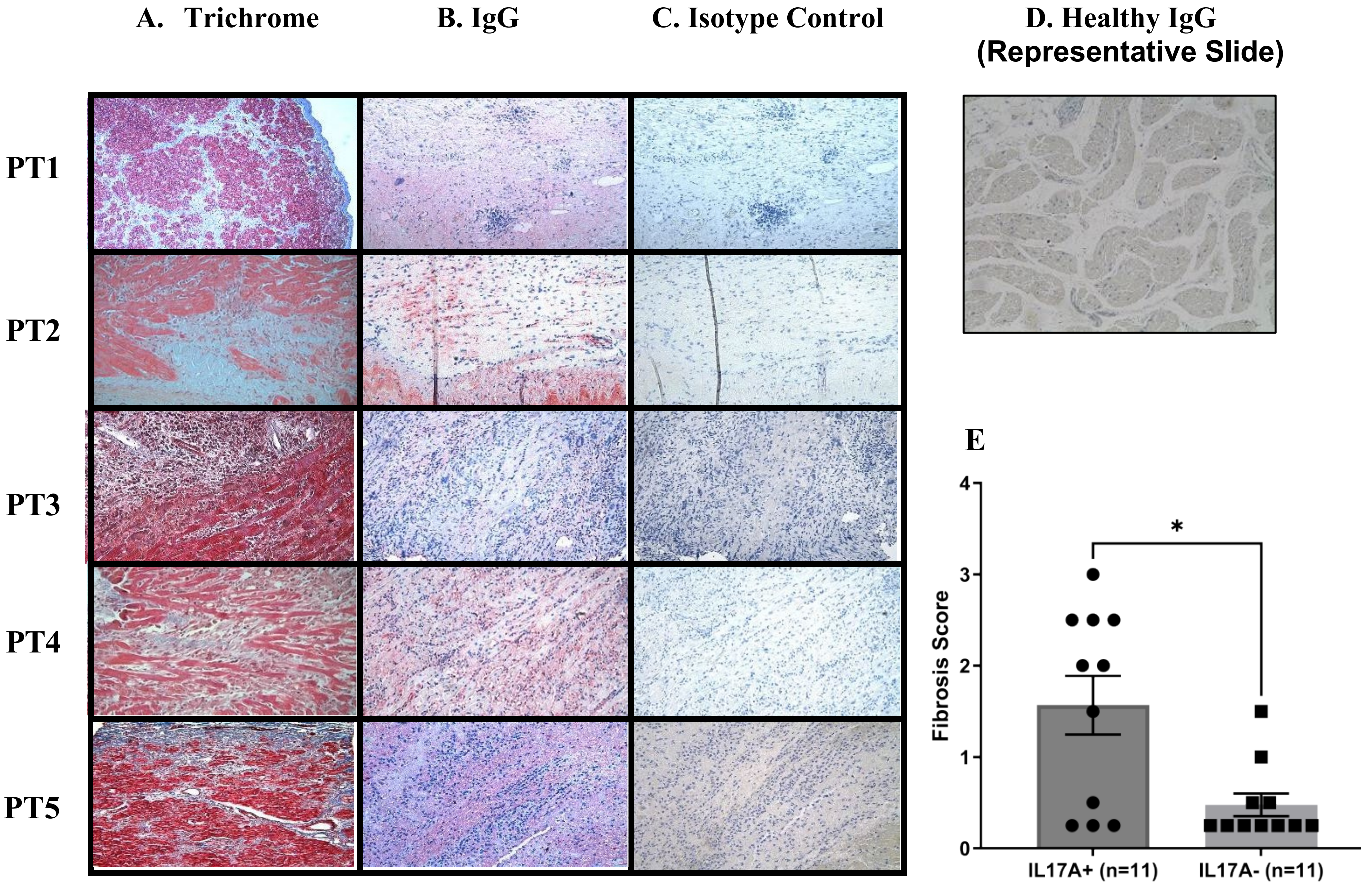

Supplementary Table 4: Antigenic Loci of Cardiac Myosin (*MYH7*) with Potential for Molecular Mimicry and Autoantibody Cross-reactivity between βARs and CM.

S2 CM peptides that were reactive to AAbs in non-recovered myocarditis/DCM patients were aligned by protein BLAST (https://blast.ncbi.nlm.nih.gov) with β1AR and β2AR amino acid sequences. We suggest three loci: **CM peptide S2-3** is a unique epitope in our non-recovered cohort and shared 62.5% identity with a sequence in the 3rd extracellular loop of the β2AR (locus 1). We observed that **CM peptide S2-17** reacted with AAbs which were also uniquely elevated in both the presently reported non-recovered patient cohort and were also elevated in our previous study of myocarditis patients (3) (locus 2). **CM peptide S2-30** contained a sequence with 46% homology with the 2nd extracellular loop of the β1AR (locus 3). See **Figure 2** in the manuscript to indicate the reactivity of each of the S2 peptides in the ELISA comparing myocarditis sera IgG reactivity with the S2 peptides to healthy sera.

Locus	MYH7 Residues	Sequence Alignment	βAR Residues
1	841-907	VQAEQDNL	295-302
		V+ QDNL	
		VHVIQDNL	
2	1066-1090	n/a	n/a
3	1234-1272	RSKAEETQRSVND	200-212
		R +E R ND	(2nd EC Loop: 197-222
		RAESDEARRCYND	

Supplementary Table 5: Other Proteins with Hypothetical Cross-Reactivity with CM Epitopes. Non-recovered myocarditis/DCM patients had elevated AAbs to S2 CM peptides. S2 peptides reactive to AAbs in our cohort were aligned to other *Homo sapiens* sequences by protein BLAST (https://blast.ncbi.nlm.nih.gov). Results were filtered by including only membrane-bound proteins present on the extracellular loop by cell expression observed in the heart*, and lastly, included only if the hypothetical sequence was a predicted epitope. ** Percent homology between the S2 CM peptide and the hypothetical second target of cross-reactive AAbs is presented below.

*Referenced from Human Protein Atlas (https://www.proteinatlas.org)
**Determined using B Cell Epitope Prediction Tool BepiPred Linear Epitope Prediction 2.0 (https://www.iedb.org)

S2 Peptide	Gene	% Homology	Predicted Epitope Site?*	Cell Expression**
S2-1	CD209	12/26(46%)	Yes	Endothelial>fibroblasts>cardiomyocytes
	Sequence	162 EKSKMQEIYQELTRLKAAVGELPEKS 187		
S2-1	ADGRE3	10/16(62%)	Yes	Monocytes > macrophages
	Sequence	129 RKELQKIVDKFESLL 143		
S2-3	FLT4	8/13(61%)	Yes	Lymph endothelial>endothelial>fibroblast
	Sequence	237 RKSLELLVGEKLV 249		
S2-3	TLR10	6/7(86%)		B cells >DC/Macrophage
	Sequence	408 LLQHKND 414		
S2-17	TLR10	9/15(60%)	Yes	B cells >DC/Macrophage
	Sequence	85 QQLDLKTFEFN 95		
S2-25	NRROS	8/12(67%	Yes	Endothelial Cells
	Sequence	122 EETAAALHA 130		
S2-29	NCAM1	5/7(71%)	Yes	Cardiomyocytes
	Sequence	617 LEGQMGE 623		
S2-29	IL20RA	9/14(64%)	Yes	Fibroblasts (low)
	Sequence	233 EKQCARTLKDQSSE 246		
S2-29	MRC2	7/8(87%)	Yes	Fibroblasts>Cardiomyocytes
	Sequence	123 CRTLGDQL 130		

Supplement References

1. Li Y, Heuser JS, Cunningham LC, Kosanke SD, Cunningham MW. Mimicry and antibody-mediated cell signaling in autoimmune myocarditis. Journal of Immunology (Baltimore, Md : 1950) 2006;177:8234-40.
2. Yu X, Patterson E, Stavrakis S et al. Development of cardiomyopathy and atrial tachyarrhythmias associated with activating autoantibodies to beta-adrenergic and muscarinic receptors. J Am Soc Hypertens 2009;3:133-40.
3. Mascaro-Blanco A, Alvarez K, Yu X et al. Consequences of unlocking the cardiac myosin molecule in human myocarditis and cardiomyopathies. Autoimmunity 2008;41:442-53.
4. Jaenicke T, Diederich KW, Haas W et al. The complete sequence of the human beta-myosin heavy chain gene and a comparative analysis of its product. Genomics 1990;8:194-206.
